# Changes in gut microbial community upon chronic kidney disease

**DOI:** 10.1371/journal.pone.0283389

**Published:** 2023-03-23

**Authors:** Wu Liu, Jiaqi Huang, Tong Liu, Yutian Hu, Kaifeng Shi, Yi Zhou, Ning Zhang

**Affiliations:** 1 Wangjing Hospital, China Academy of Chinese Medical Sciences, Beijing, China; 2 Department of Radiation Oncology, Peking University Third Hospital, Beijing, China; 3 Center of Basic Medical Research, Institute of Medical Innovation and Research, Peking University Third Hospital, Beijing, China; 4 School of Water Resources and Environment, China University of Geosciences (Beijing), Beijing, China; 5 Department of Graduate Student, Beijing University of Chinese Medicine, Beijing, China; Monash University Malaysia, MALAYSIA

## Abstract

With the increasing incidence and mortality of chronic kidney disease (CKD), targeted therapies for CKD have been explored constantly. The important role of gut microbiota on CKD has been emphasized increasingly, it is necessary to analyze the metabolic mechanism of CKD patients from the perspective of gut microbiota. In this study, bioinformatics was used to analyze the changes of gut microbiota between CKD and healthy control (HC) groups using 315 samples from NCBI database. Diversity analysis showed significant changes in evenness compared to the HC group. PCoA analysis revealed significant differences between the two groups at phylum level. In addition, the F/B ratio was higher in CKD group than in HC group, suggesting the disorder of gut microbiota, imbalance of energy absorption and the occurrence of metabolic syndrome in CKD group. The study found that compared with HC group, the abundance of bacteria associated with impaired kidney was increased in CKD group, such as *Ralstonia* and *Porphyromonas*, which were negatively associated with eGFR. PICRUSt2 was used to predict related functions and found that different pathways between the two groups were mainly related to metabolism, involving the metabolism of exogenous and endogenous substances, as well as Glycerophospholipid metabolism, which provided evidence for exploring the relationship between gut microbiota and lipid metabolism. Therefore, in subsequent studies, special attention should be paid to these bacteria and metabolic pathway, and animal experiments and metabolomics studies should be conducted explore the association between bacterial community and CKD, as well as the therapeutic effects of these microbial populations on CKD.

## Introduction

Chronic kidney disease (CKD), refers to renal function or structural abnormalities lasting for at least 3 months, which can be caused by various primary, secondary or hereditary kidney diseases. However, with the insidious onset, majority of patients with CKD are poorly recognized their disease, especially in early stages [1]. With the gradual increase in its incidence, CKD has become a major health concern worldwide [2]. A cross-sectional survey conducted in 2012 demonstrated that the prevalence of CKD in China was 10.8%, the mortality caused by CKD has been gradually increasing and it was ranked fourteenth on the list of leading causes of death [2, 3]. CKD causes a huge social burden, not only the influence of renal replacement therapy, but also the increase of global mortality caused by CKD-related cardiovascular events [2, 4]. Therefore, actively controlling factors which can contribute to the exacerbation of CKD is essential to delay the progression of CKD. Many factors had been proven to promote the progression of CKD, such as hypertension, hyperglycemia, poor control of proteinuria, and infection [5]. Apart from these typical disease course influences, the important impact of gut microbiota homeostasis on human health has been widely recognized and studied in recent years [6]. The introduction of concepts such as "Intestinal-renal syndrome" [7] and "The gut-kidney axis" [8] has opened the door to the study of gut microbiota associated with kidney disease. It has been found in several studies that the microecology of the gut microbiota, especially the structural composition and metabolites of the microbes, probably play an important role in CKD [7, 8]. With the gradually aggravated renal function, intestinal urinary toxins increase and accumulate in the blood without being timely cleared by impaired kidneys, thus aggravating the damage of kidney, destroying the intestinal barrier function and homeostasis of gut microbiota [8]. Intestinal dysbacteriosis and impairment of intestinal barrier function are two key links in the interaction between intestinal microecosystem and CKD, and as contributing factors in the progression of CKD to end-stage renal disease (ESRD) have been in focus [9].

Gut microbiome is involved in energy metabolism, contributes to the nitrogen and micronutrient homeostasis, and provides nutritional and protective functions [10, 11]. The gut microbiota can produce short-chain fatty acids (SCFA) by fermenting indigestible dietary complex carbohydrates, which is crucial for the maintenance of colonic homeostasis in healthy populations [12, 13]. However, patients with renal failure have an imbalanced intestinal ecosystem, with increased harmful aerobic bacteria and decreased beneficial anaerobic such as *Bifidobacteria* and *Lactobacillus* [14]. Furthermore, urea hydrolysis processes that correspond to the community composition dynamics raise intestinal ammonia and ammonium hydroxide [15]. Ammonia accumulation has been reported to disrupt the intestinal epithelial barrier and function, causing the development of systemic inflammation in CKD patients [10, 15]. All these lead to an increase in intestinal pH, which in turn can cause mucosal irritation and negatively affect the growth of commensal bacteria [16]. The intestinal epithelial barrier function depends on the effects of probiotics and commensal bacteria, and the composition of the gut microbiota in CKD patients has undergone a change from a symbiotic to dysbiotic state [16]. When progressing to ESRD, an increase in bacterial families with urease, uricase, indole and p-cresol-forming enzymes can be observed, and the flora containing enzymes that convert dietary fiber into SCFA are decreasing, aggravating the accumulation of toxins in intestine, severely decreasing the mucosal barrier integrity, disrupting immune tolerance, and promoting endotoxemia and systemic inflammation [10, 15]. Apart from the changes and feedback effects of gut microbiota caused by CKD, drug use and dietary restrictions can also lead to changes in the gut microbiota. CKD patients need to control potassium intake, leading to a lower intake of potassium-rich fruits and vegetables, which may in turn lead to inadequate intake of dietary fibers, the dominant substrate for bacterial fermentation. Insufficient dietary fibers intake then prolonged colonic transit time and inducing an imbalance of glycolytic bacteria and proteolytic bacteria, eventually converting the flora metabolism from glycolytic to protein fermentation pattern [17].

In addition, some gut microbiota metabolites, such as Indoxyl sulfate (IS), p-Cresyl sulfate (pCS) and trimethylamine-nitrogen oxide (TMAO), have been reported to impair endothelial function in CKD patients, and to be involved in the development and progression of CKD and cardiovascular complications by causing inflammation and oxidative stress [18]. The content of IS and pCS in early CKD patients begins to rise [19]. As protein-bound uremic retention solutes, IS and pCS are difficult to be cleared via binding to albumin by hemodialysis (HD) [20]. In addition to being associated with the deterioration of CKD, elevated levels of TMAO also indicate a poor prognosis, the increased cardiovascular risk and premature death in patients with CKD [9]. Intervention of intestinal microecology has a certain effect on delaying the progression of CKD. Enterotoxin sorbents [21], probiotics [22], and fecal flora transplantation [23] can reduce serum toxins and improve the state of microinflammation in patients with CKD. As adjuvants, probiotics and prebiotics have beneficial effects on removing uremic toxins. Several studies have shown that taking probiotics and prebiotics to regulate the intestinal environment and microbiota of CKD patients can reduce blood urea nitrogen levels in CKD stages 3–4 [14], and decline the uremic toxins as well as increase beneficial bacteria counts in dialysis patients [24, 25].

Even though the role of gut microbiota homeostasis of CKD has been recognized to some extent. Due to the lack of relevant studies and samples, the patterns of the gut microbiota composition associated with the course of CKD remain unclear. Investigating the composition characteristics and classification of intestinal communities in CKD patients can provide a theoretical basis for subsequent targeted treatment. Moreover, the discovery of new beneficial bacteria as supplements can improve the intestinal homeostasis of CKD, reduce the accumulation of endotoxin, as well as delay the deterioration of CKD. Changes in dominant flora caused by alterations in bacterial community compositions lead to the difference in microorganism functions. While the correlation between microbiota and pathways needs further study. With the development of technologies, “smart” bacteria may guide the immune system, metabolism pathways, and so on. Comprehensively understanding the functions and pathways of human microbiota to design personal medicine programs according to specific microbiota for CKD patients is also a core issue [26]. To answer these questions, we integrated studies related to the gut microbiota of CKD patients and the corresponding amplicon sequencing data from NCBI database. We analyzed the gut microbiota associated with the course of CKD by comparing the composition patterns and abundance characteristics between CKD patients and healthy individuals. The effect of CKD on the structure of gut microbiota community was explored by amplicon sequences, and the differences in dominant species of gut microbiota with and without CKD were elucidated. Phylogenetic Investigation of Communities by Reconstruction of Unobserved States (PICRUSt2) was used to predict the major functions differences and underlying metabolic process to improve awareness of the structure and characteristics of gut microbiota in patients with CKD, as well as pharmacological interventions to regulate the abundance and diversity of gut microbiota.

## Materials and methods

### Acquisition of sequence information and Bioinformatics Analysis

**Fig 1:**
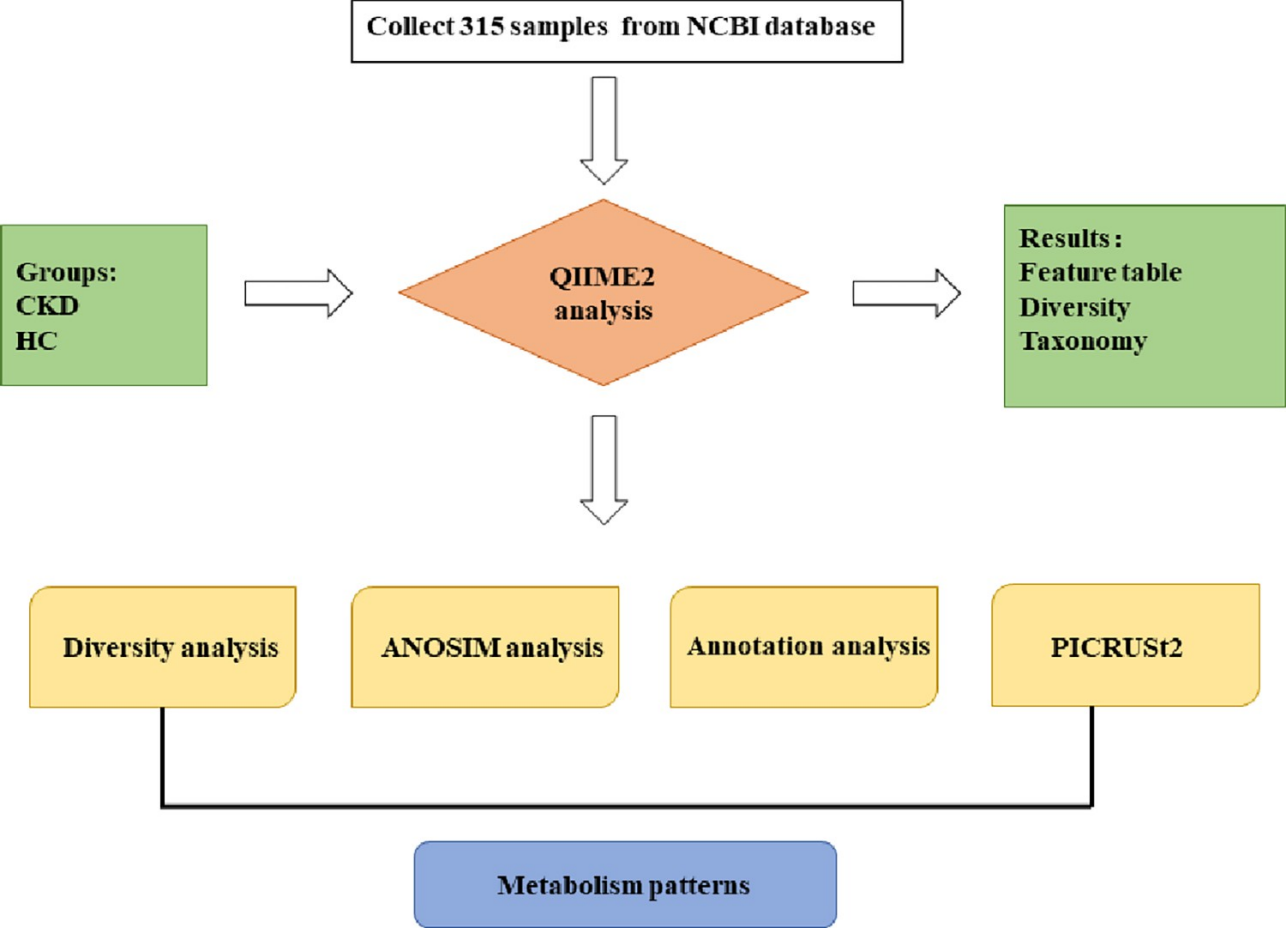
The schematic representation of bioinformatic analysis.

A total of 315 gut microbiota samples from patients with CKD and healthy control (HC) were retrieved from SRA database of NCBI (https://www.ncbi.nlm.nih.gov/sra), the schematic representation of bioinformatic analysis was presented in Fig 1. All sequences were from V3-V4 regions of 16S rRNA. Collecting the metadata from relevant literature, and the samples were divided into CKD and HC groups, the detail was in S1 Table. QIIME2 2022.2 [27] was used to perform the bioinformatics of gut microbiota. Raw sequence data from MiniSeq and HiSeq platforms (paired-end) were quality filtered using the Demux plugin and then denoised with DADA2 [28]. The Shannon and ACE indexes of alpha diversity were compared using Welch Two Sample t-test, meantime beta diversity was analyzed via estimating the Principal Coordinate Analysis (PCoA) by using Bray–Curtis dissimilarity matrix. And phylogeny tree construction was using fasttree2 [29]. Using Bayesian SILVA 138 Full Classifier to annotate species [30]. Finally, the Kyoto Encyclopedia of Genes and Genomes (KEGG) pathway analysis of gut microbiota was performed by PICRUSt2 [31]. However, it should be noted that the prediction results of PICRUSt2 were limited. And R 4.0.4 software was used to analyze the data in this study, the ALDEx2 package in R was used to compare the gut microbiota between the two group.

## Results and discussion

### Analysis of gut microbiota diversity

**Fig 2:**
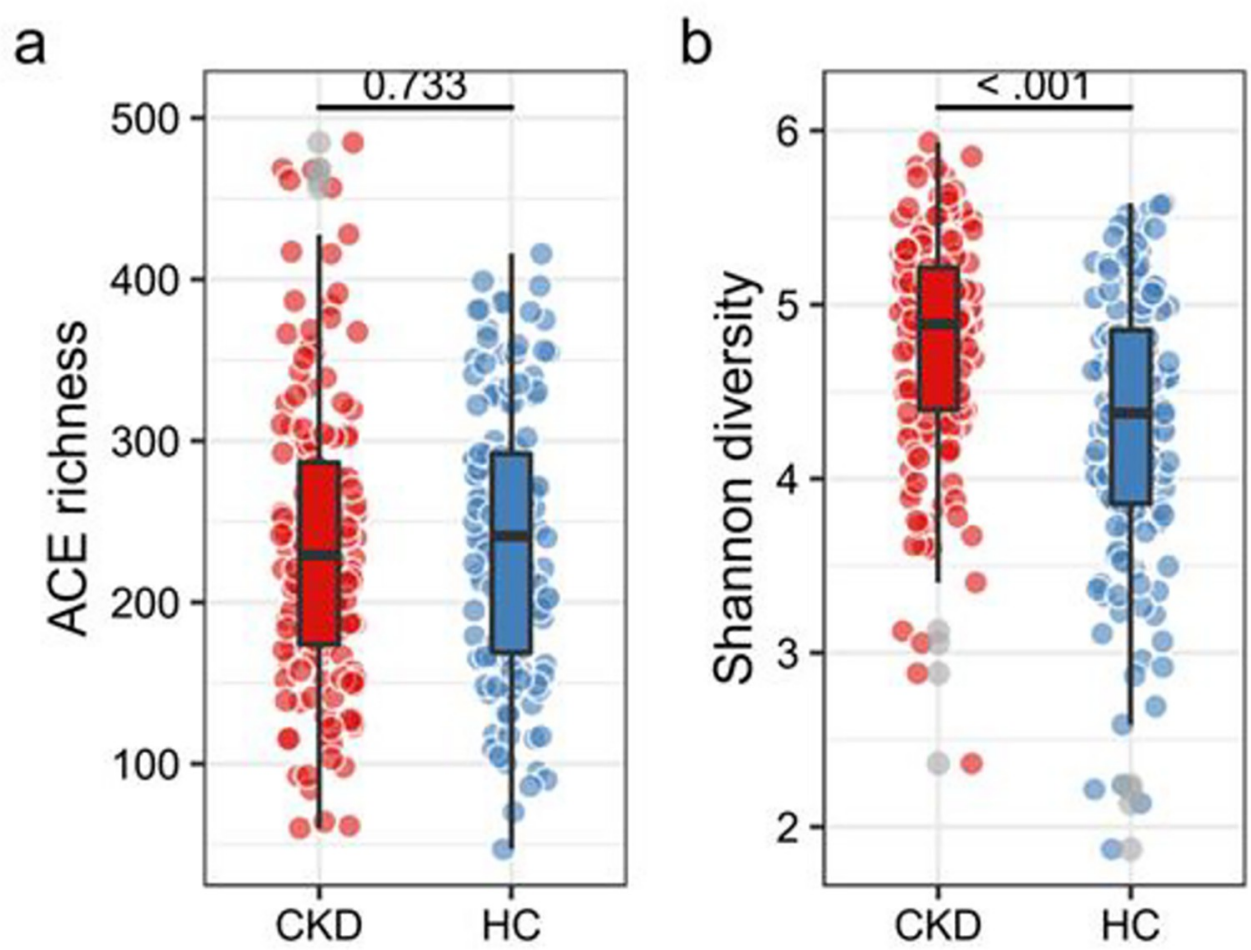
The Alpha diversity of gut microbiota in different groups. (a) ACE index reflected the richness of two groups, it showed that the influence of different groups on microorganisms richness might be limited (P = 0.733), (b) The results showed that Shannon index between two groups was statistically different (P < 0.001), indicating that the evenness of the gut microbiota was affected by CKD.

After denoising by DADA2, a total of 298 samples were obtained, and more than 4 million sequences were obtained, with an average of 13531 sequences per sample. The alpha diversity of the microbiota could be divided into richness and evenness. The ACE index reflected the richness of samples while the Shannon index considered both richness and evenness. The Welch test was performed on ACE richness index to analyze differences in microbiota richness in different groups, and the following results were obtained (Fig 2A). The Welch Two Sample t-test testing the difference of ACE by group (mean in group CKD = 235.65, mean in group HC = 239.08) suggested that the effect was negative, statistically not significant, and very small (difference = -3.43, 95% CI [-23.16, 16.31], t (293.97) = -0.34, p = 0.733; Cohen’s d = -0.04, 95% CI [-0.27, 0.19]). Therefore, the influence of different groups on microorganisms richness might be limited. Welch test was further performed on Shannon index, and the results showed in Fig 2B presented that the difference of Shannon by group (mean in group CKD = 4.77, mean in group HC = 4.30) suggested that the effect was positive, statistically significant, and medium (difference = 0.47, 95% CI [0.31, 0.64], t (271.82) = 5.73, p < .001; Cohen’s d = 0.70, 95% CI [0.45, 0.94]). These results suggested that CKD significantly improved the diversity of gut microbiota. Since only the Shannon index but not ACE index was significantly changed, we could infer that CKD mainly affected the evenness of the gut microbiota, resulting in a change in the species distribution within community, which might be associated with progressive renal failure. Elevated serum urea caused by renal failure could be converted into ammonia in gastrointestinal tract, contributing to overgrowth of bacterial family with a tendency of urea metabolism and further impairing endothelial function in CKD patients [10, 18].

**Fig 3:**
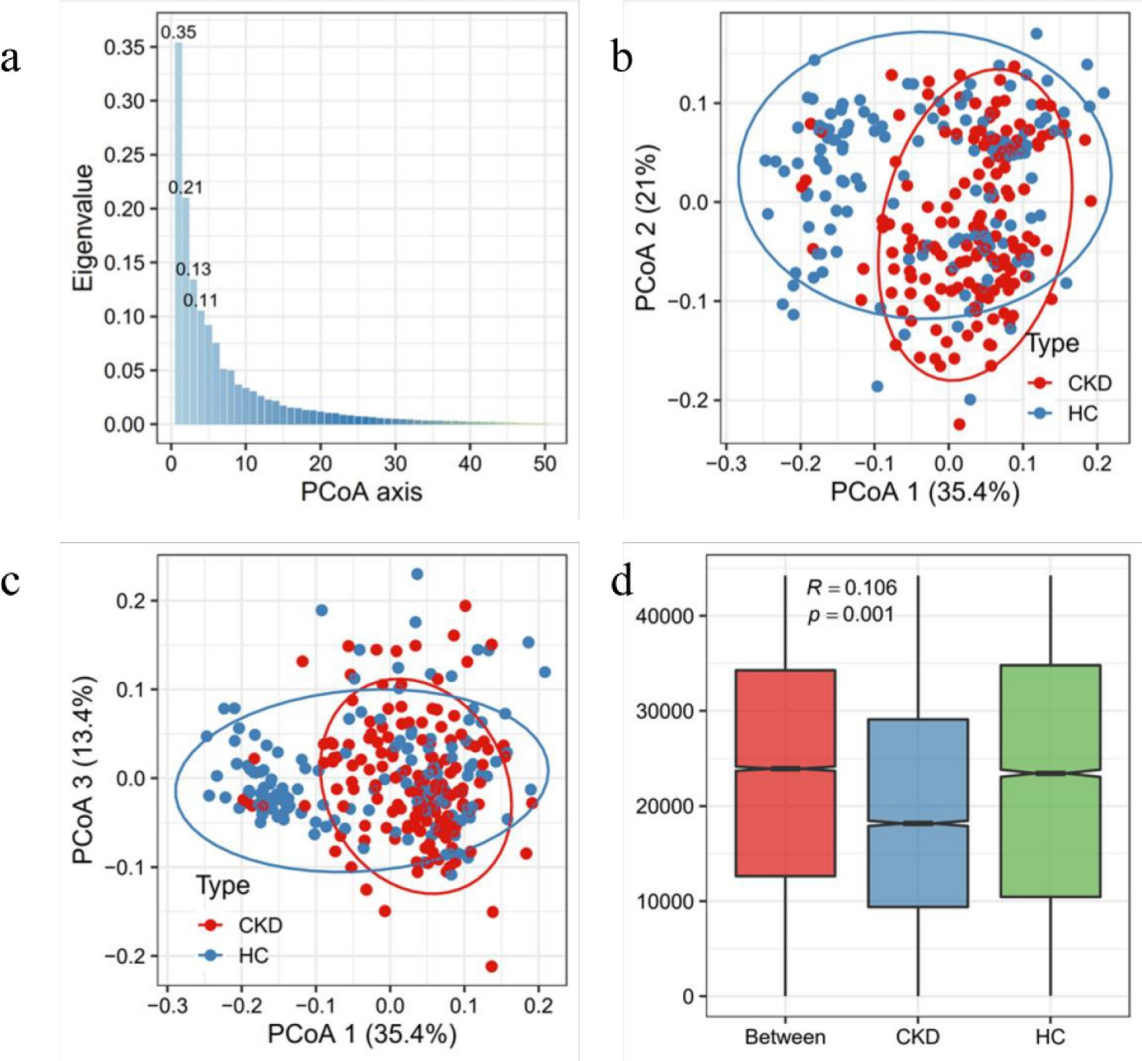
PCoA analysis in phylum level in different groups. (a) PCoA scree plot showed the first two axes account for 56% of the total variance. (b) PCoA ordination plot of axis 1 and 2, its showed that the samples were relatively separated at axis 1. (c) PCoA ordination plot of axis 1 and 3, it showed that there was no visual separation on axis 3. (d) ANOSIM test exhibited the intergroup differences in two groups were greater than the intragroup group (P = 0.001).

PCoA was run at the phylum level, the abundance of phylum was first calculated, and then log transformed for the calculation of Bray–Curtis dissimilarity matrix (Note for the performance of log transformation, a pseudo 1 was first add to the abundance counts). As shown in Fig 3A, the scree plot analyzed by PCoA analysis indicated that the first two axes represent 56% of the total variance, and the third axis represents 13% of the total variance. As shown in Fig 3B and 3C, the samples were relatively separated at axis 1 of the PCoA, with slight separation at axis 2, but almost no visual separation on axis 3, although axis 3 explained 13.4% variance between the samples. In addition, PCoA plot of the different platforms was shown in S1 Fig.

As shown in Fig 3D, analysis of Similarities (ANOSIM) results presented that intergroup differences in HC and CKD groups were greater than the intragroup difference (p<0.05). This demonstrated that sample separation in the PCoA plot was not only visual, but also statistically significant. Further analysis was performed using adonis2 function of the vegan package to perform permutational MANOVA on the data with 999 permutations. The results displayed that the groups of samples were statistically significant, and the null hypothesis of the same groups could be rejected.

**Fig 4:**
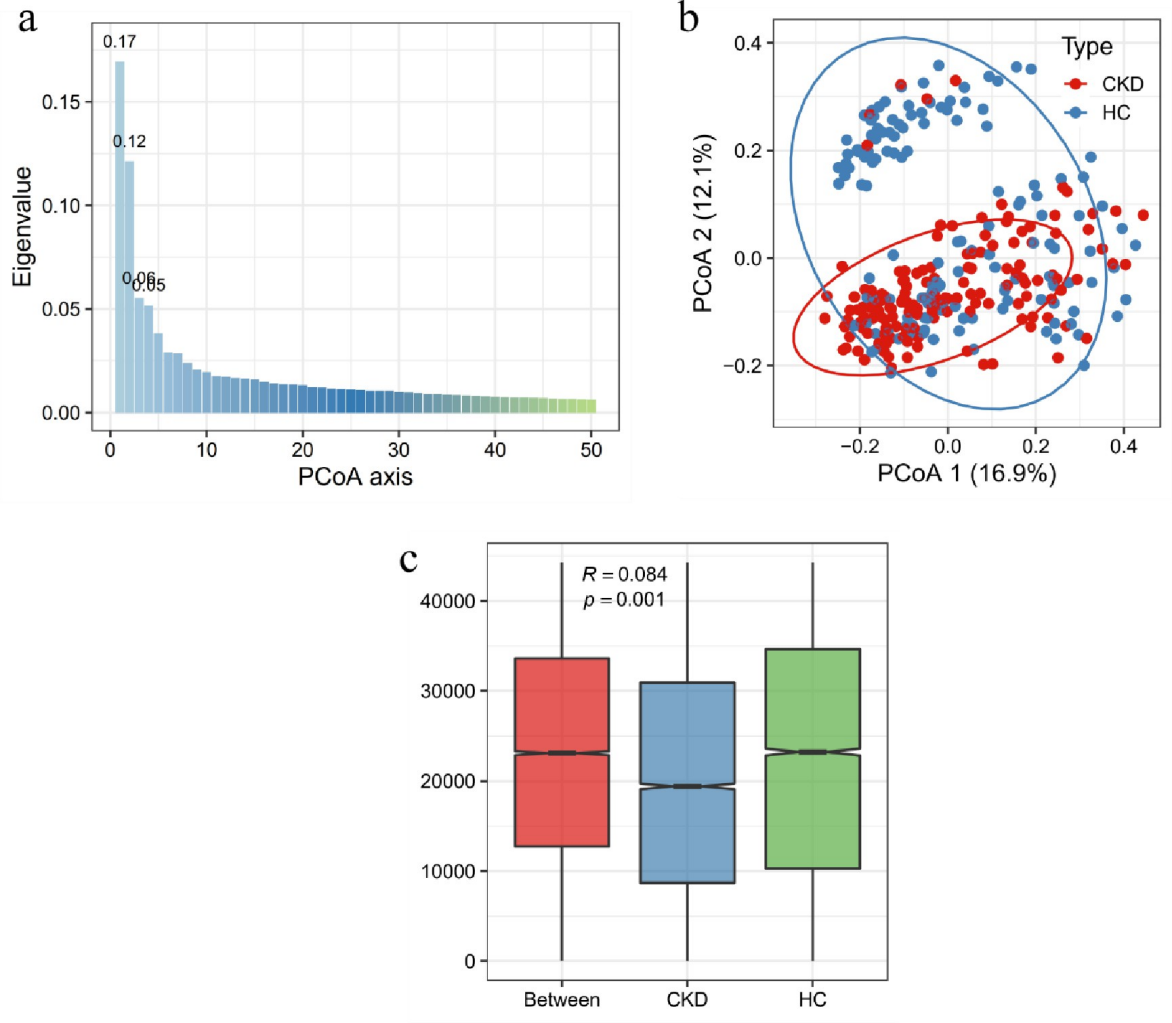
PCoA analysis in genus level in different groups. (a) PCoA scree plot showed the first two axes account for 29% of the total variance. (b) PCoA ordination plot of axis 1 and 2 showed the clustering of samples in different groups. (c) ANOSIM test showed the intergroup difference between CKD and HC groups (P = 0.001).

Similarly, we also analyzed PCoA at the genus level, as shown in Fig 4a, the scree plot exhibited the first two axes represent 29% of the total variance. The samples were separated at the axis 1 of PCoA, accounting for 16.9% variance. However, Fig 4b was presented that samples in HC group were visibly divided into two parts. This might be related to the data collected from different countries and genders. Differences in diet structure or living habits among healthy people from different regions might have influenced the distribution of gut microbiota. In contrast, CKD samples exhibited tight clustering. We inferred that this may be related to an increase in endotoxin-producing microbiota due to changes in intestinal environment, which transformed the microbiota from a complex, homogeneous and coordinated community to one that simpler but more dominant [32]. Therefore, we also used ANOSIM similarity analysis on the data at genus level. As shown in Fig 4C, the intergroup difference was still statistically significant (p<0.05), although R was lower indicating greater intragroup variation in the HC group.

### Annotation analysis of gut microbiota species

**Fig 5:**
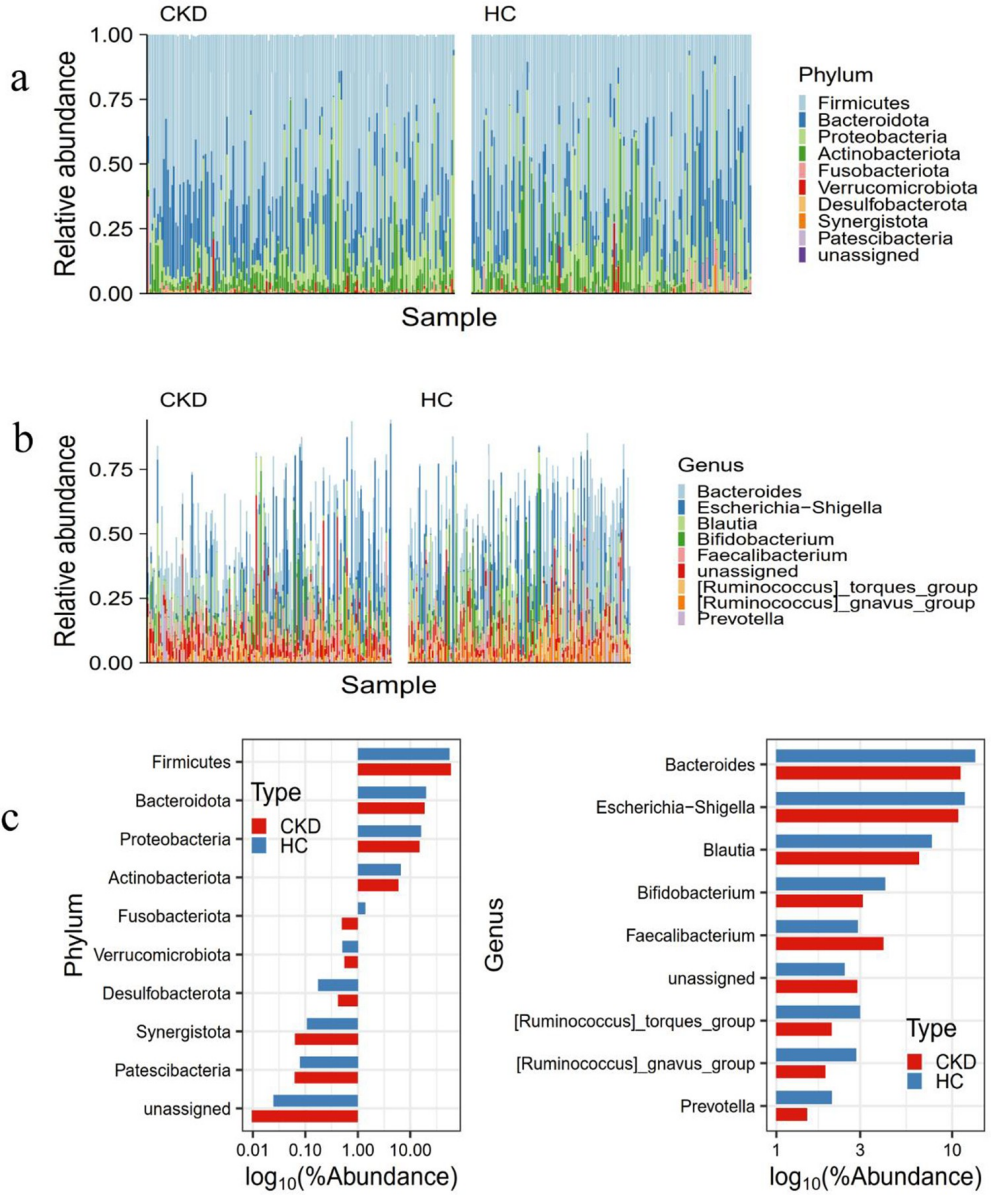
Annotation analysis of gut microbiota species. (a) bar plot of the top 10 phyla with the highest abundance, (b) bar plot of the top 10 genera with the highest abundance, (c) average relative abundance of the top 10 phyla or genera in abundance.

According to the results of species annotation, the species of each sample were analyzed by the taxonomy of the phylum and genus level. As shown in Fig 5, species differences among samples and the proportions can be understood from the bar chart. The top 10 phyla were *Firmicutes*, *Bacteroidota*, *Proteobacteria*, *Actinobacteriota*, *Fusobacteriota*, *Verrucomicrobiota*, *Desulfobacterota*, *Synergistota*, and *Patescibacteria*. Consistent with the literature [33], *Firmicutes*, *Bacteroidota*, *Proteobacteria*, and *Actinobacteriota* were the dominant bacterial phyla in the two groups. Among them, the proportions of *Firmicutes* and *Bacteroidota* in two groups were shown in Table 1.

**Table 1:** F/B ratio in CKD and HC groups.

CKD		HC	
Phylum	Proportion	Phylum	Proportion
***Firmicute***	55.49%	***Firmicute***	51.87%
***Bacteroidota***	13.63%	***Bacteroidota***	26.93%
**F/B**	4.07		1.93

**Fig 6:**
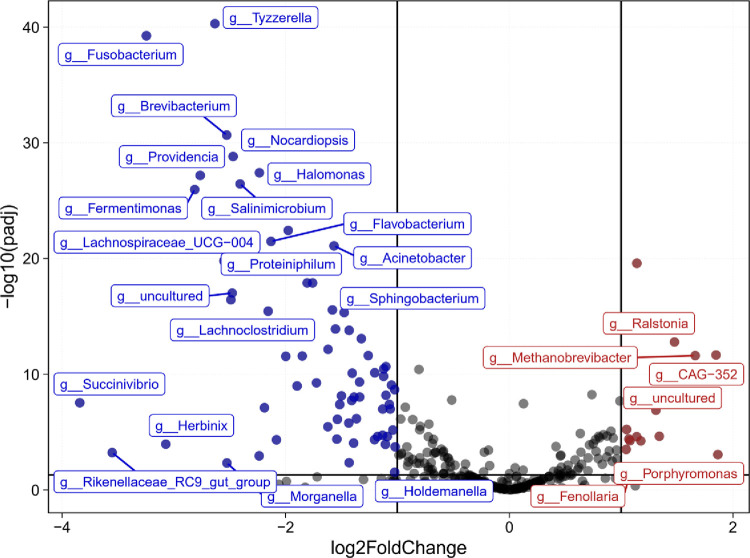
Volcano map of the genus-level microbial communities with different abundance in the CKD group compared with HC group. Most of the bacteria with decreased abundance belonged to Firmicutes, Bacteroidota, Proteobacteria, and Actinobacteriota.

In the human gut microbiota, *Firmicutes* and *Bacteroidota* were the two most abundant phyla. They played a crucial role in regulating the host inflammation and immune balance [34]. The change of the ratio of *Firmicutes* and *Bacteroidota* (F/B) in the microbial community was an important indicator, it reflected the disorder of gut microbiota, and the increase of F/B ratio was positively correlated with intestinal permeability [35, 36]. As shown in Table 1, the F/B ratios in CKD and HC groups were 4.07 and 1.93, respectively, suggesting the dysregulation of gut microbiota in CKD patients. Changes in energy absorption caused by the imbalance of the proportion of these two phyla resulted in metabolic syndrome and exacerbation of CKD [33]. Fig 6 showed that compared with HC group, the abundances of *Methanobrevibacter*, *Ralstonia*, *Fenollaria*, *Porphyromonas* in CKD group were increased. Inversely, the abundances of *Tyzzerella*, *Fusobacterium*, *Fusobacteria*, *Brevibacterium*, *Nocardiopsis*, *Providencia*, *Halomonas*, *Fermentimonas*, *Salinimicrobium*, *Flavobacterium*, *Acinetobacter*, *Lachnospiraceae_UCG-004*, *Succinivibrio*, *Herbinix*, *Morganella*, *Holdemanella* were decreased. Besides, most of the bacteria with decreased abundance belonged to *Firmicutes*, *Bacteroidota*, *Proteobacteria*, and *Actinobacteriota*.

### Regulatory mechanism prediction of gut microbiota in CKD

In order to explore how gut microbiota regulate CKD, functional composition predictions and annotations were made using PICRUSt2 based on KEGG database. The ALDEx2 package was used to extract different pathways, and a total of 176 pathways were obtained in S2 Table, which were divided into six categories, including Metabolism, Human Diseases, Organismal Systems, Genetic Information Processing, Environmental Information Processing, and Cellular Processes. According to we.eBH <0.05, we got 78 different pathways. The top 10 significantly different pathway between the two groups were displayed in Table 2, which were mainly associated with metabolism. And the abundances of these metabolism pathways in CKD group were decreased. Among them, current studies had known that Chloroalkane and chloroalkene degradation, Glycerophospholipid metabolism were related to the deterioration of renal function [37, 38].

**Table 2:** TOP10 different pathways.

ko	Level 1 pathway	Level 3 pathway	We.eBH
**ko05131**	Human Diseases	Shigellosis	1.76E-12
**ko00625**	Metabolism	Chloroalkane and chloroalkene degradation	9.70E-08
**ko00983**	Metabolism	Drug metabolism—other enzymes	5.30E-07
**ko00643**	Metabolism	Styrene degradation	2.60E-06
**ko02010**	Environmental Information Processing	ABC transporters	3.26E-05
**ko00791**	Metabolism	Atrazine degradation	3.73E-05
**ko00960**	Metabolism	Tropane, piperidine and pyridine alkaloid biosynthesis	3.98E-05
**ko00633**	Metabolism	Nitrotoluene degradation	7.65E-05
**ko00361**	Metabolism	Chlorocyclohexane and chlorobenzene degradation	0.000135
**ko00564**	Metabolism	Glycerophospholipid metabolism	0.000246

## Discussion

A study demonstrated that the disturbance of gut microbiota was interrelated with CKD. In the early stage of CKD, both the composition and metabolic activity of gut microbiota began to change, simultaneously, the imbalance of gut microbiota was a risk factor for the CKD progression and various complications [39]. The increase of urea level and the proliferation of urease bacteria in patients with CKD led to an accumulation of ammonium in the gastrointestinal tract, raising the intestinal pH and weakening the junctions of intestinal cells, ultimately altering the permeability of the intestinal mucosa [40]. In the course of CKD, the alteration of intestinal barrier, intestinal permeability, and gut bacterial community contributed to disruption of gut epithelial barrier complexes so that endotoxins and other harmful substances could flow into systemic circulation, inducing the occurrence of systemic inflammation, further promoting the release of pro-inflammatory cytokines and exacerbating CKD [40]. Changes in the composition, abundance and functional gene differences of human intestinal microecology were vital factors that could exacerbate the progression of diseases in the body. Alterations of the gut microbiota at various phases of CKD were also focus of current research. It is important to explore the relationship between gut microbiota and CKD, and investigate the difference of gut microbiota between patients with CKD and healthy people, as well as the different microbiota that could aggravate disease progression. Thereby adjusting the use of probiotics, prebiotics and synbiotics according to the distribution characteristics of floras at different phases has become a crucial measure to delay the course of CKD to ESRD. By comparing the gut microbiota and intestinal metabolites of different stages of CKD patients and healthy participants, Chen et al. verified that in contrast to the healthy group, unique bacterial aggregation was found in the fecal samples of CKD patients at different stages, and the abundance of *Streptococcus*, *Klebsiella pneumonia*, and *Haemophilus parainfluenzae* in different stages of CKD patients was relatively high [41]. Moreover, the relative abundance of *Klebsiella pneumonia* could be used as a marker for CKD progression, and the metabolites, such as S-adenosylhomocysteine, L-Carnitine, Propionic acid, and Myristic acid in fecal were gradually increased across the exacerbation of CKD [41]. A Chinese study elucidated the characteristics of gut microbiota in non-dialysis patients with CKD stages 2–5 [42]. Compared with CKD stages 3–5, dominant bacterial phyla in CKD2 were more evenly distributed, and the abundance of *Bifidobacteria* was high, and the abundances of *Faecalibacterium*, *Escherichia-Shigella*, and *Ruminococcus* were higher than those in CKD stages 2–4, despite the difference was not statistically significant [42]. The level of protein-bound uremic toxin in plasma also increased with the progression of CKD, and the removal of pCS, p-cresyl glucuronide, IS was decreased due to the impaired kidney, resulting in an overall solute removal disorder, although this might be affected by multiple factors, modulating the gut microbiota could still reduce the accumulation of uremic toxins [43]. Besides, changes in gut microbiota caused by different renal replacement therapies have also been expounded. Crespo-Salgado et al. demonstrated that a significant increase in *Proteobacteria* in patients undergoing peritoneal dialysis (PD) via comparing the gut microbiota of pediatric patients undergoing HD and PD [44]. And compared with healthy participants, the abundance of *Enterobacteriaceae* in PD patients was increased, which was related to the intestinal absorption of glucose from dialysate, the increase of *Enterobacteriaceae* might lead to the risk of peritonitis infection in PD patients [44]. As previously stated, as the main source of urinary toxin accumulation in patients with CKD, the study of gut microbiota had been brought into focus.

In this study, we analyzed the gut microbiota of CKD patients and healthy participants by bioinformatics analysis. Higher Shannon index but comparable ACE index were found in CKD group compared to HC group, indicating that CKD process mainly changed evenness but not richness of gut microbiota. These results were consistent with the results from Vaziri et al. [10] and Hida et al. [45]. According to estimated glomerular filtration rate (eGFR), Kim et al. [46] classified CKD patients before dialysis and analyzed the gut microbiota of each subgroup, they indicated that changes in diversity of gut microbiota were associated with uremia progression. Research on the different species between CKD and HC groups could help to delay the progression of renal function and improve the gut-kidney axis via modulating beneficial bacteria in the intestine. An increased F/B ratio has been widely recognized as the marker of intestinal dysbiosis [36]. In this study, the F/B ratio in CKD group was increased compared with HC group, it demonstrated that intestinal microbiome disorder and abnormal energy metabolism occurred in CKD group. *Firmicutes* and *Bacteroidota* were involved in the production of SCFA in intestine, fermenting food into butyrate and propionate, and there was a symbiotic relationship between *Firmicutes* and *Bacteroidota* that could jointly promote host to absorb or store energy [47]. *Firmicutes* mainly helped the host to absorb energy from the diet [48]. A growing body of evidence showed that *Firmicutes* was positively correlated with gut microbiota dysbiosis, and it could convert polysaccharides into SCFA and monosaccharides, resulting in more energy absorption, eventually causing obesity and insulin resistance [49, 50]. Colonized in the human distal intestine, *Bacteroides* were able to provide nutrients for human host via degrading and fermenting multiple polysaccharides and host-derived glycoconjugates (glycans), maintaining the normal physiological function of the intestine [48, 51]. And the concentrations of SCFA were significantly associated with *Bacteroidetes*, polysaccharides were fermented in the distal gastrointestinal tract under anaerobic conditions to produce SCFA, providing energy for colonic epithelial cells [18]. With declining kidney function, the accumulated nitrogenous metabolic waste products such as circulating urea and creatinine spread to the gastrointestinal tract, then disrupted microorganisms associated with SCFAs production. Impaired production of SCFAs, especially butyrate, further contributed to a lack of energy sources for colon cells and damage to intestinal integrity [52]. The increase of proteolytic bacteria, which might lead to an increase in serum urea, creatine, and toxic compounds such as indoles, phenols and ammonia, was also associated with disease-propagating and pro-inflammatory pathways [38, 52].

In addition, as previously mentioned, we analyzed the differential bacterial between the two groups and found that consistent with current literature, the relative abundances of *Ralstonia* and *Porphyromonas*, which were negatively correlated with eGFR, increased in CKD group [53, 54]. *Ralstonia* was associated with increased endotoxin levels, ultimately resulting in systemic endotoxins and chronic inflammation [55]. Expression of KIM-1 was significantly increased in kidney organoids treated with *Ralstonia*, and tubular damage was observed in those treated with *Ralstonia pickettii* [53]. *Porphyromonas* was a genus of *Bacteroidota*, among which *Porphyromonas gingivalis* was revealed associated with periodontal disease and CKD [54]. This study also found that the relative abundance of *Fenollaria* in CKD group was higher than HC group. Most studies were focused on *Fenollaria massiliensis*, which is close with infection [56]. There was still a lack of studies on *Fenollaria* and CKD, the correlation between *Fenollaria* and infection might be related to the systemic inflammatory state of CKD. In addition, the increased abundance of *Methanobrevibacter* might be associated with its properties. *Methanobrevibacter* could regulate the specificity of polysaccharide fermentation and affect calories stored in fat, it had gas-producing properties, the increase in relative abundance could lead to severe flatulence [57, 58]. The decreased renal function in CKD patients resulted in reduced excretion of metabolites. Accumulated metabolic wastes in digestive tract and stimulated the intestinal mucosal, eventually causing abdominal distension, nausea, vomiting and other digestive tract symptoms. These symptoms in CKD patients might be related to the increase of *Methanobrevibacter*. *Firmicutes*, *Bacteroidota*, *Proteobacteria*, and *Actinobacteriota* account for 98% of the human gut microbiota, and the reduction of these microbiota was crucial for the disturbance of intestinal microecology and the occurrence of diseases [59]. In this study, we found that compared with HC group, most decreased bacteria abundance in CKD group belonged to *Firmicutes* and *Bacteroidota*, which were the dominant bacteria producing SCFA (such as butyrate, propionate and acetate). *Fusobacterium*, one of the decreased bacteria in CKD group, was associated with the production of SCFA, especially butyrate [60]. And studies showed that shifted abundance of *Lachnospiraceae* family was a strong indication of gut health, *Lachnospiraceae* might convert the deoxy sugars rhamnose and fucose into propionate and propanol through propanediol pathway [38, 61]. Moreover, we also found the abundance of *Holdemanella* was reduced, it is contrary to the results that *Holdemanella* was positive with the progression of CKD pointed out by Lun et al. [62]. Therefore, the specific role of *Holdemanella* still needed to be further explored. However, we must admit the deficiencies. Since the original data of renal function indicators of patients were not accessible, we cannot perform a correlation analysis of differential bacterial and renal function. This study still provided some evidence for the alterations of CKD gut microbiota, while the regulation of these differential bacteria needed to be verified by basic research in the future. Similarly, the relationship between differential bacteria and impaired kidney, and the composition of the microbiota at different stages of CKD should be studied, and understanding the diversity, the alterations of abundance and evenness could provide bases for the subsequent clinical targeted therapies of related bacteria.

According to the functional prediction analysis of the gut microbiota in two groups based on level 1 and level 3, of which, level 1 was most related to metabolism, and level 3 clearly indicated the degradation of various metabolites, such as Styrene, Chloroalkane and chloroalkene, Atrazine, Nitrotoluene, Chlorocyclohexane and chlorobenzene degradation. As a metabolic organ, kidney could excrete exogenous substances, such as Chloroalkane and chloroalkene [37], Nitrotoluene [63], Chlorocyclohexane and chlorobenzen [64]. Liu et al. indicated that Chloroalkane and chloroalkene degradation pathway could predict the morbidity of CKD [37], with the decline of renal function, impaired glomerular filtration led to the accumulation of toxins in metabolite excretion disorders which aggravated the progression of CKD. Besides, Atrazine degradation pathway was mainly used to remove excess or unwanted bacteria in the intestinal tract [65]. With the decrease of eGFR in CKD patients, the floras which could produce uremic toxin precursors such as indoles, p-cresol and trimethylamine (TMA) increased, leading to an increase in IS, pCS, TMAO, and reactive oxygen species (ROS), inducing renal fibrosis [66]. Whether the Atrazine degradation pathway could play a role in eliminating harmful floras in intestine was still worth exploring. In addition, glycerophospholipid was the main component of cell membranes and played an important role in cell signal transmission. Glycerophospholipid metabolism was the most significantly altered pathway in CKD rats, and the increase in glycerophospholipid levels was inversely associated with eGFR [67]. However, it should be noted that PICRUSt2 was used to analyze the pathway inference of the data in this study, and the functional pathway association between gut microbiota and CKD should still be verified by animal experiments or human fecal metabolomics.

## Conclusion

In this study, bioinformatics was used to analyze the gut microbiota of patients in CKD and HC groups, and the species annotation, diversity analysis and functional prediction analysis in two groups were performed. The results showed that the F/B ratio in CKD group was higher than that in HC group, confirming that the gut microbiota in CKD patients was disturbed, and further indicating that gut microbiota was involved in the occurrence and development of CKD. Consistent with disease progression, increased abundances of *Ralstonia* and *Porphyromonas* were found in CKD patients, which were considered associated with worse kidney injury and poor eGFR. Therefore, *Ralstonia* and *Porphyromonas* may be indicators of the progression of CKD. In addition, 8 of the top10 main differential pathways were concentrated in metabolism, including the metabolism of a variety of endogenous and exogenous substances through the kidney. Correspondingly, many differential bacteria abundances, such as *Methanobrevibacter*, *Fusobacterium* and *Lachnospiraceae*, may affect host metabolism such as lipid metabolism, which is consistent with the concentration of differential metabolic pathways, suggesting a vital link between metabolism and gut microbiota in the pathogenesis of CKD. The above also provided a basis for the follow-up study to explore the connection between gut microbiota and lipid metabolism in CKD. Based on the above, we conclude the following hypothesis, gut microbiota of CKD patients is dislocated, with significant evenness changes, and may affect the excretion of uremic toxins via regulating the metabolism of endogenous and exogenous substances through kidney, thus affecting the progression of CKD. Further animal experiments and human fecal metabolomics studies were needed for verification.

## Supporting information

S1 Fig PCoA analysis based on the sequencing platforms. (DOCX)

S1 Table Metadata of sequence information. (DOCX)

S2 Table Differential pathways of gut microbiota between CKD and HC groups. (DOCX)
