# Supplementary figures and images for: Changes in gut microbial community upon chronic kidney disease
Source: PLoS One. 2023 Mar 23;18(3):e0283389. doi: 10.1371/journal.pone.0283389 (PMC10035866; doi:10.1371/journal.pone.0283389)

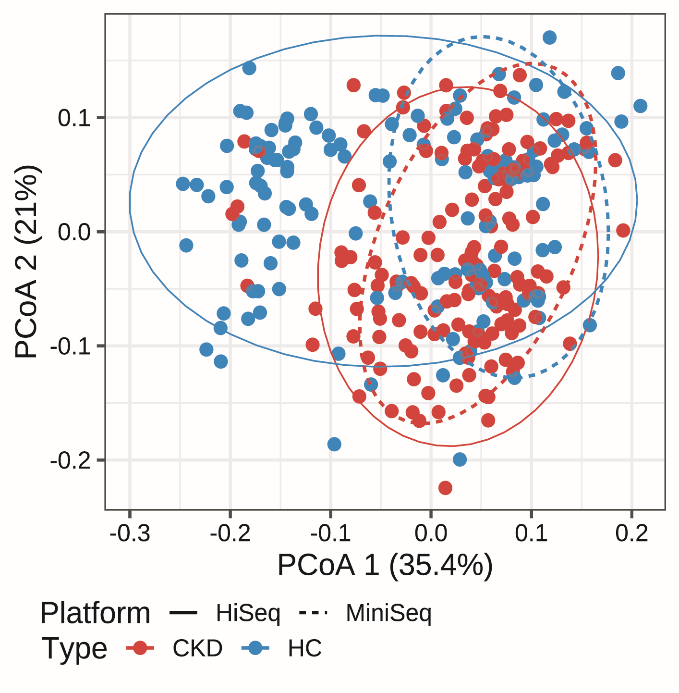


**S1 Fig. PCoA analysis based on the sequencing platforms.**

Supplement: S1 Fig — (DOCX) [file pone.0283389.s001.docx]
