# Supplementary material for: Changes in gut microbial community upon chronic kidney disease
Source: PLoS One. 2023 Mar 23;18(3):e0283389. doi: 10.1371/journal.pone.0283389 (PMC10035866; doi:10.1371/journal.pone.0283389)
Supplement: S1 Table — (DOCX) [file pone.0283389.s002.docx]

**S1_Table. Metadata of sequence information**

| **SRA_ID** | **Disease** | **Country** | **Sex** | **Platform** | **Reference** |  |
| --- | --- | --- | --- | --- | --- | --- |
| **SRR12534021** | HC | China | NA | HiSeq | [1] | |
| **SRR12534022** | HC | China | NA | HiSeq | [1] | |
| **SRR12534023** | HC | China | NA | HiSeq | [1] | |
| **SRR12534024** | HC | China | NA | HiSeq | [1] | |
| **SRR12534025** | HC | China | NA | HiSeq | [1] | |
| **SRR12534026** | HC | China | NA | HiSeq | [1] | |
| **SRR12534027** | HC | China | NA | HiSeq | [1] | |
| **SRR12534028** | CKD | China | NA | HiSeq | [1] | |
| **SRR12534029** | HC | China | NA | HiSeq | [1] | |
| **SRR12534030** | HC | China | NA | HiSeq | [1] | |
| **SRR12534031** | HC | China | NA | HiSeq | [1] | |
| **SRR12534032** | HC | China | NA | HiSeq | [1] | |
| **SRR12534033** | HC | China | NA | HiSeq | [1] | |
| **SRR12534034** | HC | China | NA | HiSeq | [1] | |
| **SRR12534035** | HC | China | NA | HiSeq | [1] | |
| **SRR12534036** | HC | China | NA | HiSeq | [1] | |
| **SRR12534037** | HC | China | NA | HiSeq | [1] | |
| **SRR12534038** | HC | China | NA | HiSeq | [1] | |
| **SRR12534039** | CKD | China | NA | HiSeq | [1] | |
| **SRR12534040** | HC | China | NA | HiSeq | [1] | |
| **SRR12534041** | HC | China | NA | HiSeq | [1] | |
| **SRR12534042** | HC | China | NA | HiSeq | [1] | |
| **SRR12534043** | HC | China | NA | HiSeq | [1] | |
| **SRR12534044** | HC | China | NA | HiSeq | [1] | |
| **SRR12534045** | HC | China | NA | HiSeq | [1] | |
| **SRR12534046** | HC | China | NA | HiSeq | [1] | |
| **SRR12534047** | HC | China | NA | HiSeq | [1] | |
| **SRR12534048** | HC | China | NA | HiSeq | [1] | |
| **SRR12534049** | HC | China | NA | HiSeq | [1] | |
| **SRR12534050** | CKD | China | NA | HiSeq | [1] | |
| **SRR12534051** | HC | China | NA | HiSeq | [1] | |
| **SRR12534052** | HC | China | NA | HiSeq | [1] | |
| **SRR12534053** | HC | China | NA | HiSeq | [1] | |
| **SRR12534054** | HC | China | NA | HiSeq | [1] | |
| **SRR12534055** | HC | China | NA | HiSeq | [1] | |
| **SRR12534056** | HC | China | NA | HiSeq | [1] | |
| **SRR12534057** | HC | China | NA | HiSeq | [1] | |
| **SRR12534058** | HC | China | NA | HiSeq | [1] | |
| **SRR12534059** | HC | China | NA | HiSeq | [1] | |
| **SRR12534060** | HC | China | NA | HiSeq | [1] | |
| **SRR12534061** | CKD | China | NA | HiSeq | [1] | |
| **SRR12534062** | HC | China | NA | HiSeq | [1] | |
| **SRR12534063** | HC | China | NA | HiSeq | [1] | |
| **SRR12534064** | HC | China | NA | HiSeq | [1] | |
| **SRR12534065** | HC | China | NA | HiSeq | [1] | |
| **SRR12534066** | HC | China | NA | HiSeq | [1] | |
| **SRR12534067** | HC | China | NA | HiSeq | [1] | |
| **SRR12534068** | HC | China | NA | HiSeq | [1] | |
| **SRR12534069** | HC | China | NA | HiSeq | [1] | |
| **SRR12534070** | HC | China | NA | HiSeq | [1] | |
| **SRR12534071** | HC | China | NA | HiSeq | [1] | |
| **SRR12534072** | CKD | China | NA | HiSeq | [1] | |
| **SRR12534073** | HC | China | NA | HiSeq | [1] | |
| **SRR12534074** | HC | China | NA | HiSeq | [1] | |
| **SRR12534075** | HC | China | NA | HiSeq | [1] | |
| **SRR12534076** | HC | China | NA | HiSeq | [1] | |
| **SRR12534077** | HC | China | NA | HiSeq | [1] | |
| **SRR12534078** | HC | China | NA | HiSeq | [1] | |
| **SRR12534079** | HC | China | NA | HiSeq | [1] | |
| **SRR12534080** | HC | China | NA | HiSeq | [1] | |
| **SRR12534081** | HC | China | NA | HiSeq | [1] | |
| **SRR12534082** | HC | China | NA | HiSeq | [1] | |
| **SRR12534083** | CKD | China | NA | HiSeq | [1] | |
| **SRR12534084** | HC | China | NA | HiSeq | [1] | |
| **SRR12534085** | HC | China | NA | HiSeq | [1] | |
| **SRR12534086** | HC | China | NA | HiSeq | [1] | |
| **SRR12534087** | HC | China | NA | HiSeq | [1] | |
| **SRR12534088** | HC | China | NA | HiSeq | [1] | |
| **SRR12534089** | HC | China | NA | HiSeq | [1] | |
| **SRR12534090** | HC | China | NA | HiSeq | [1] | |
| **SRR12534091** | HC | China | NA | HiSeq | [1] | |
| **SRR12534092** | CKD | China | NA | HiSeq | [1] | |
| **SRR12534093** | HC | China | NA | HiSeq | [1] | |
| **SRR12534094** | CKD | China | NA | HiSeq | [1] | |
| **SRR12534095** | CKD | China | NA | HiSeq | [1] | |
| **SRR12534096** | CKD | China | NA | HiSeq | [1] | |
| **SRR12534097** | CKD | China | NA | HiSeq | [1] | |
| **SRR12534098** | CKD | China | NA | HiSeq | [1] | |
| **SRR12534099** | CKD | China | NA | HiSeq | [1] | |
| **SRR12534100** | CKD | China | NA | HiSeq | [1] | |
| **SRR12534101** | CKD | China | NA | HiSeq | [1] | |
| **SRR12534102** | CKD | China | NA | HiSeq | [1] | |
| **SRR12534103** | CKD | China | NA | HiSeq | [1] | |
| **SRR12534104** | CKD | China | NA | HiSeq | [1] | |
| **SRR12534105** | CKD | China | NA | HiSeq | [1] | |
| **SRR12534106** | CKD | China | NA | HiSeq | [1] | |
| **SRR12534107** | CKD | China | NA | HiSeq | [1] | |
| **SRR12534108** | CKD | China | NA | HiSeq | [1] | |
| **SRR12534109** | CKD | China | NA | HiSeq | [1] | |
| **SRR12534110** | CKD | China | NA | HiSeq | [1] | |
| **SRR12534111** | CKD | China | NA | HiSeq | [1] | |
| **SRR12534112** | CKD | China | NA | HiSeq | [1] | |
| **SRR12534113** | CKD | China | NA | HiSeq | [1] | |
| **SRR12534114** | CKD | China | NA | HiSeq | [1] | |
| **SRR12534115** | CKD | China | NA | HiSeq | [1] | |
| **SRR12534116** | CKD | China | NA | HiSeq | [1] | |
| **SRR12534117** | CKD | China | NA | HiSeq | [1] | |
| **SRR12534118** | CKD | China | NA | HiSeq | [1] | |
| **SRR12534119** | CKD | China | NA | HiSeq | [1] | |
| **SRR12534120** | CKD | China | NA | HiSeq | [1] | |
| **SRR12534121** | CKD | China | NA | HiSeq | [1] | |
| **SRR12534122** | CKD | China | NA | HiSeq | [1] | |
| **SRR12534123** | CKD | China | NA | HiSeq | [1] | |
| **SRR12534124** | CKD | China | NA | HiSeq | [1] | |
| **SRR12534125** | CKD | China | NA | HiSeq | [1] | |
| **SRR12534126** | CKD | China | NA | HiSeq | [1] | |
| **SRR12534127** | CKD | China | NA | HiSeq | [1] | |
| **SRR12534128** | CKD | China | NA | HiSeq | [1] | |
| **SRR12534129** | CKD | China | NA | HiSeq | [1] | |
| **SRR12534130** | CKD | China | NA | HiSeq | [1] | |
| **SRR12534131** | CKD | China | NA | HiSeq | [1] | |
| **SRR12534132** | CKD | China | NA | HiSeq | [1] | |
| **SRR12534133** | CKD | China | NA | HiSeq | [1] | |
| **SRR12534134** | CKD | China | NA | HiSeq | [1] | |
| **SRR12534135** | CKD | China | NA | HiSeq | [1] | |
| **SRR12534136** | CKD | China | NA | HiSeq | [1] | |
| **SRR12534137** | CKD | China | NA | HiSeq | [1] | |
| **SRR12534138** | CKD | China | NA | HiSeq | [1] | |
| **SRR12534139** | CKD | China | NA | HiSeq | [1] | |
| **SRR12534140** | CKD | China | NA | HiSeq | [1] | |
| **SRR12534141** | CKD | China | NA | HiSeq | [1] | |
| **SRR12534142** | CKD | China | NA | HiSeq | [1] | |
| **SRR12534143** | CKD | China | NA | HiSeq | [1] | |
| **SRR12534144** | CKD | China | NA | HiSeq | [1] | |
| **SRR12534145** | CKD | China | NA | HiSeq | [1] | |
| **SRR12534146** | CKD | China | NA | HiSeq | [1] | |
| **SRR12534147** | CKD | China | NA | HiSeq | [1] | |
| **SRR12534148** | CKD | China | NA | HiSeq | [1] | |
| **SRR12534149** | CKD | China | NA | HiSeq | [1] | |
| **SRR12534150** | CKD | China | NA | HiSeq | [1] | |
| **SRR12534151** | CKD | China | NA | HiSeq | [1] | |
| **SRR12534152** | CKD | China | NA | HiSeq | [1] | |
| **SRR12534153** | CKD | China | NA | HiSeq | [1] | |
| **SRR12534154** | CKD | China | NA | HiSeq | [1] | |
| **SRR12534155** | CKD | China | NA | HiSeq | [1] | |
| **SRR12534156** | CKD | China | NA | HiSeq | [1] | |
| **SRR12534157** | CKD | China | NA | HiSeq | [1] | |
| **SRR12534158** | CKD | China | NA | HiSeq | [1] | |
| **SRR12534159** | CKD | China | NA | HiSeq | [1] | |
| **SRR12534160** | CKD | China | NA | HiSeq | [1] | |
| **SRR12534161** | CKD | China | NA | HiSeq | [1] | |
| **SRR12534162** | CKD | China | NA | HiSeq | [1] | |
| **SRR12534163** | CKD | China | NA | HiSeq | [1] | |
| **SRR12534164** | CKD | China | NA | HiSeq | [1] | |
| **SRR12534165** | CKD | China | NA | HiSeq | [1] | |
| **SRR12534166** | CKD | China | NA | HiSeq | [1] | |
| **SRR12534167** | CKD | China | NA | HiSeq | [1] | |
| **SRR12534168** | CKD | China | NA | HiSeq | [1] | |
| **SRR12534169** | CKD | China | NA | HiSeq | [1] | |
| **SRR12534170** | CKD | China | NA | HiSeq | [1] | |
| **SRR12534171** | CKD | China | NA | HiSeq | [1] | |
| **SRR12534172** | CKD | China | NA | HiSeq | [1] | |
| **SRR12534173** | CKD | China | NA | HiSeq | [1] | |
| **SRR12534174** | CKD | China | NA | HiSeq | [1] | |
| **SRR12534175** | CKD | China | NA | HiSeq | [1] | |
| **SRR12534176** | HC | China | NA | HiSeq | [1] | |
| **SRR12534177** | HC | China | NA | HiSeq | [1] | |
| **SRR12534178** | HC | China | NA | HiSeq | [1] | |
| **SRR12534179** | HC | China | NA | HiSeq | [1] | |
| **SRR12534180** | HC | China | NA | HiSeq | [1] | |
| **SRR12534181** | HC | China | NA | HiSeq | [1] | |
| **SRR12534182** | HC | China | NA | HiSeq | [1] | |
| **SRR12534183** | CKD | China | NA | HiSeq | [1] | |
| **SRR12534184** | HC | China | NA | HiSeq | [1] | |
| **SRR12534185** | HC | China | NA | HiSeq | [1] | |
| **SRR12534186** | HC | China | NA | HiSeq | [1] | |
| **SRR12534187** | HC | China | NA | HiSeq | [1] | |
| **SRR12534188** | HC | China | NA | HiSeq | [1] | |
| **SRR12534189** | HC | China | NA | HiSeq | [1] | |
| **SRR12534190** | HC | China | NA | HiSeq | [1] | |
| **SRR12534191** | HC | China | NA | HiSeq | [1] | |
| **SRR12534192** | HC | China | NA | HiSeq | [1] | |
| **SRR12534193** | HC | China | NA | HiSeq | [1] | |
| **SRR12534194** | CKD | China | NA | HiSeq | [1] | |
| **SRR12534195** | HC | China | NA | HiSeq | [1] | |
| **SRR12534196** | HC | China | NA | HiSeq | [1] | |
| **SRR12534197** | HC | China | NA | HiSeq | [1] | |
| **SRR12534198** | HC | China | NA | HiSeq | [1] | |
| **SRR12534199** | HC | China | NA | HiSeq | [1] | |
| **SRR12534200** | HC | China | NA | HiSeq | [1] | |
| **SRR12534201** | HC | China | NA | HiSeq | [1] | |
| **SRR12534202** | HC | China | NA | HiSeq | [1] | |
| **SRR12534203** | HC | China | NA | HiSeq | [1] | |
| **SRR12534204** | HC | China | NA | HiSeq | [1] | |
| **SRR12534205** | CKD | China | NA | HiSeq | [1] | |
| **SRR12534206** | HC | China | NA | HiSeq | [1] | |
| **SRR12534207** | HC | China | NA | HiSeq | [1] | |
| **SRR12534208** | HC | China | NA | HiSeq | [1] | |
| **SRR12534209** | HC | China | NA | HiSeq | [1] | |
| **SRR12534210** | HC | China | NA | HiSeq | [1] | |
| **SRR12534211** | HC | China | NA | HiSeq | [1] | |
| **SRR12534212** | HC | China | NA | HiSeq | [1] | |
| **SRR12534213** | HC | China | NA | HiSeq | [1] | |
| **SRR12534214** | HC | China | NA | HiSeq | [1] | |
| **SRR12534215** | HC | China | NA | HiSeq | [1] | |
| **SRR12534216** | CKD | China | NA | HiSeq | [1] | |
| **SRR12534217** | CKD | China | NA | HiSeq | [1] | |
| **SRR12534218** | HC | China | NA | HiSeq | [1] | |
| **SRR12534219** | HC | China | NA | HiSeq | [1] | |
| **SRR12534220** | HC | China | NA | HiSeq | [1] | |
| **SRR12534221** | HC | China | NA | HiSeq | [1] | |
| **SRR12534222** | HC | China | NA | HiSeq | [1] | |
| **SRR12534223** | HC | China | NA | HiSeq | [1] | |
| **SRR12534224** | HC | China | NA | HiSeq | [1] | |
| **SRR12534225** | HC | China | NA | HiSeq | [1] | |
| **SRR12534226** | HC | China | NA | HiSeq | [1] | |
| **SRR12534227** | HC | China | NA | HiSeq | [1] | |
| **SRR12534228** | CKD | China | NA | HiSeq | [1] | |
| **SRR12534229** | HC | China | NA | HiSeq | [1] | |
| **SRR12534230** | HC | China | NA | HiSeq | [1] | |
| **SRR12534231** | HC | China | NA | HiSeq | [1] | |
| **SRR12534232** | HC | China | NA | HiSeq | [1] | |
| **SRR12534233** | HC | China | NA | HiSeq | [1] | |
| **SRR12534234** | HC | China | NA | HiSeq | [1] | |
| **SRR12534235** | HC | China | NA | HiSeq | [1] | |
| **SRR12534236** | HC | China | NA | HiSeq | [1] | |
| **SRR12534237** | HC | China | NA | HiSeq | [1] | |
| **SRR12534238** | HC | China | NA | HiSeq | [1] | |
| **SRR12534239** | CKD | China | NA | HiSeq | [1] | |
| **SRR12534240** | HC | China | NA | HiSeq | [1] | |
| **SRR12534241** | HC | China | NA | HiSeq | [1] | |
| **SRR12534242** | HC | China | NA | HiSeq | [1] | |
| **SRR12534243** | HC | China | NA | HiSeq | [1] | |
| **SRR12534244** | HC | China | NA | HiSeq | [1] | |
| **SRR12534245** | HC | China | NA | HiSeq | [1] | |
| **SRR12534246** | HC | China | NA | HiSeq | [1] | |
| **SRR12534247** | HC | China | NA | HiSeq | [1] | |
| **SRR12534248** | HC | China | NA | HiSeq | [1] | |
| **SRR12534249** | HC | China | NA | HiSeq | [1] | |
| **SRR12534250** | CKD | China | NA | HiSeq | [1] | |
| **SRR12534251** | HC | China | NA | HiSeq | [1] | |
| **SRR12534252** | HC | China | NA | HiSeq | [1] | |
| **SRR12534253** | HC | China | NA | HiSeq | [1] | |
| **SRR12534254** | HC | China | NA | HiSeq | [1] | |
| **SRR12534255** | CKD | China | NA | HiSeq | [1] | |
| **SRR12534256** | CKD | China | NA | HiSeq | [1] | |
| **SRR12534257** | CKD | China | NA | HiSeq | [1] | |
| **ERR3761021** | CKD | Italy | Male | MiniSeq | [2] | |
| **ERR3761022** | CKD | Italy | Male | MiniSeq | [2] | |
| **ERR3761023** | CKD | Italy | Male | MiniSeq | [2] | |
| **ERR3761024** | CKD | Italy | Male | MiniSeq | [2] | |
| **ERR3761025** | CKD | Italy | Femal | MiniSeq | [2] | |
| **ERR3761026** | CKD | Italy | Male | MiniSeq | [2] | |
| **ERR3761027** | CKD | Italy | Male | MiniSeq | [2] | |
| **ERR3761028** | CKD | Italy | Femal | MiniSeq | [2] | |
| **ERR3761029** | CKD | Italy | Male | MiniSeq | [2] | |
| **ERR3761030** | CKD | Italy | Male | MiniSeq | [2] | |
| **ERR3761031** | CKD | Italy | Male | MiniSeq | [2] | |
| **ERR3761032** | CKD | Italy | Male | MiniSeq | [2] | |
| **ERR3761033** | CKD | Italy | Femal | MiniSeq | [2] | |
| **ERR3761034** | CKD | Italy | Male | MiniSeq | [2] | |
| **ERR3761035** | CKD | Italy | Femal | MiniSeq | [2] | |
| **ERR3761036** | CKD | Italy | Femal | MiniSeq | [2] | |
| **ERR3761037** | CKD | Italy | Male | MiniSeq | [2] | |
| **ERR3761038** | CKD | Italy | Femal | MiniSeq | [2] | |
| **ERR3761039** | CKD | Italy | Male | MiniSeq | [2] | |
| **ERR3761040** | CKD | Italy | Male | MiniSeq | [2] | |
| **ERR3761041** | CKD | Italy | Male | MiniSeq | [2] | |
| **ERR3761042** | CKD | Italy | Male | MiniSeq | [2] | |
| **ERR3761043** | CKD | Italy | Male | MiniSeq | [2] | |
| **ERR3761044** | CKD | Italy | Male | MiniSeq | [2] | |
| **ERR3761045** | CKD | Italy | Femal | MiniSeq | [2] | |
| **ERR3761046** | CKD | Italy | Femal | MiniSeq | [2] | |
| **ERR3761047** | CKD | Italy | Male | MiniSeq | [2] | |
| **ERR3761048** | CKD | Italy | Male | MiniSeq | [2] | |
| **ERR3761049** | CKD | Italy | Male | MiniSeq | [2] | |
| **ERR3761050** | CKD | Italy | Femal | MiniSeq | [2] | |
| **ERR3761051** | CKD | Italy | Male | MiniSeq | [2] | |
| **ERR3761052** | CKD | Italy | Male | MiniSeq | [2] | |
| **ERR3761053** | CKD | Italy | Femal | MiniSeq | [2] | |
| **ERR3761054** | CKD | Italy | Femal | MiniSeq | [2] | |
| **ERR3761055** | CKD | Italy | Male | MiniSeq | [2] | |
| **ERR3761056** | CKD | Italy | Male | MiniSeq | [2] | |
| **ERR3761057** | CKD | Italy | Male | MiniSeq | [2] | |
| **ERR3761058** | CKD | Italy | Femal | MiniSeq | [2] | |
| **ERR3761059** | CKD | Italy | Male | MiniSeq | [2] | |
| **ERR3761060** | CKD | Italy | Femal | MiniSeq | [2] | |
| **ERR3761061** | CKD | Italy | Male | MiniSeq | [2] | |
| **ERR3761062** | CKD | Italy | Femal | MiniSeq | [2] | |
| **ERR3761063** | CKD | Italy | Male | MiniSeq | [2] | |
| **ERR3761064** | CKD | Italy | Femal | MiniSeq | [2] | |
| **ERR3761065** | CKD | Italy | Femal | MiniSeq | [2] | |
| **ERR3761066** | CKD | Italy | Male | MiniSeq | [2] | |
| **ERR3761067** | CKD | Italy | Femal | MiniSeq | [2] | |
| **ERR3761068** | CKD | Italy | Male | MiniSeq | [2] | |
| **ERR3761069** | CKD | Italy | Male | MiniSeq | [2] | |
| **ERR3761070** | CKD | Italy | Male | MiniSeq | [2] | |
| **ERR3761071** | CKD | Italy | Femal | MiniSeq | [2] | |
| **ERR3761072** | CKD | Italy | Male | MiniSeq | [2] | |
| **ERR3761073** | CKD | Italy | Femal | MiniSeq | [2] | |
| **ERR3761074** | CKD | Italy | Male | MiniSeq | [2] | |
| **ERR3761075** | CKD | Italy | Male | MiniSeq | [2] | |
| **ERR3761076** | CKD | Italy | Male | MiniSeq | [2] | |
| **ERR3761077** | CKD | Italy | Male | MiniSeq | [2] | |
| **ERR3761078** | CKD | Italy | Male | MiniSeq | [2] | |
| **ERR3761079** | CKD | Italy | Femal | MiniSeq | [2] | |
| **ERR3761080** | CKD | Italy | Male | MiniSeq | [2] | |
| **ERR3761081** | CKD | Italy | Male | MiniSeq | [2] | |
| **ERR3761082** | CKD | Italy | Male | MiniSeq | [2] | |
| **ERR3761083** | CKD | Italy | Male | MiniSeq | [2] | |
| **ERR3761084** | HC | Italy | Male | MiniSeq | [2] | |
| **ERR3761085** | HC | Italy | Femal | MiniSeq | [2] | |
| **ERR3761086** | HC | Italy | Femal | MiniSeq | [2] | |
| **ERR3761087** | HC | Italy | Male | MiniSeq | [2] | |
| **ERR3761088** | HC | Italy | Male | MiniSeq | [2] | |
| **ERR3761089** | HC | Italy | Femal | MiniSeq | [2] | |
| **ERR3761090** | HC | Italy | Femal | MiniSeq | [2] | |
| **ERR3761091** | HC | Italy | Femal | MiniSeq | [2] | |
| **ERR3761092** | HC | Italy | Male | MiniSeq | [2] | |
| **ERR3761093** | HC | Italy | Femal | MiniSeq | [2] | |
| **ERR3761094** | HC | Italy | Femal | MiniSeq | [2] | |
| **ERR3761095** | HC | Italy | Femal | MiniSeq | [2] | |
| **ERR3761096** | HC | Italy | Femal | MiniSeq | [2] | |
| **ERR3761097** | HC | Italy | Femal | MiniSeq | [2] | |
| **ERR3761098** | HC | Italy | Femal | MiniSeq | [2] | |

**Reference:**

[1] Liu F, Xu X, Chao L, et al. Alteration of the Gut Microbiome in Chronic Kidney Disease Patients and Its Association With Serum Free Immunoglobulin Light Chains. Front Immunol. 2021 Apr 1;12:609700. doi: 10.3389/fimmu.2021.609768.

[2] Margiotta E, Miragoli F, Callegari ML, et al. Gut microbiota composition and frailty in elderly patients with Chronic Kidney Disease. PLoS One. 2020 Apr 1;15(4):e0228530. doi: 10.1371/journal.pone.0228583.
