# Supplementary material for: Changes in gut microbial community upon chronic kidney disease
Source: PLoS One. 2023 Mar 23;18(3):e0283389. doi: 10.1371/journal.pone.0283389 (PMC10035866; doi:10.1371/journal.pone.0283389)
Supplement: S2 Table — (DOCX) [file pone.0283389.s003.docx]

**S2 Table. Differential pathways of gut microbiota between CKD and HC groups**

| **ko** | **rab.all** | **rab.win.CKD** | **rab.win.HC** | **diff.btw** | **diff.win** | **effect** | **overlap** | **we.ep** | **we.eBH** | **wi.ep** | **wi.eBH** |
| --- | --- | --- | --- | --- | --- | --- | --- | --- | --- | --- | --- |
| **ko00640** | 4.568888 | 4.49955 | 4.659683 | 0.139162 | 0.525399 | 0.176801 | 0.389322 | 0.854898 | 0.875317 | 0.003183 | 0.008357 |
| **ko00280** | 3.560475 | 3.529329 | 3.602353 | 0.032383 | 0.503363 | 0.060174 | 0.475105 | 0.769513 | 0.800532 | 0.448479 | 0.489101 |
| **ko00350** | 3.194475 | 3.139129 | 3.262579 | 0.101635 | 0.487594 | 0.180353 | 0.415517 | 0.767728 | 0.798157 | 0.014307 | 0.025551 |
| **ko00941** | -2.81001 | -2.87178 | -2.7562 | 0.06172 | 2.354742 | 0.019699 | 0.4872 | 0.759912 | 0.79182 | 0.699307 | 0.731645 |
| **ko00908** | 4.30672 | 4.286844 | 4.333973 | 0.026899 | 0.688071 | 0.037669 | 0.482304 | 0.758065 | 0.789628 | 0.658194 | 0.693161 |
| **ko00930** | 0.123245 | -0.01241 | 0.337289 | 0.362771 | 2.630548 | 0.109064 | 0.4388 | 0.756378 | 0.788965 | 0.103789 | 0.134137 |
| **ko00430** | 4.485735 | 4.41348 | 4.572863 | 0.121619 | 0.553356 | 0.124882 | 0.4072 | 0.754025 | 0.787166 | 0.00671 | 0.014573 |
| **ko00780** | 5.594783 | 5.59315 | 5.597151 | -0.00055 | 0.622788 | -0.00094 | 0.4994 | 0.730055 | 0.767179 | 0.799815 | 0.825329 |
| **ko00627** | 2.071384 | 2.001568 | 2.1518 | 0.167698 | 0.723021 | 0.181999 | 0.4006 | 0.706019 | 0.744158 | 0.006901 | 0.014632 |
| **ko05146** | -1.63438 | -1.61514 | -1.65722 | -0.01353 | 1.314267 | -0.00759 | 0.496501 | 0.691572 | 0.729813 | 0.747882 | 0.777245 |
| **ko05143** | -2.16619 | -2.49025 | -1.81562 | 0.539988 | 3.030974 | 0.124694 | 0.422316 | 0.693416 | 0.728335 | 0.028897 | 0.04553 |
| **ko00360** | 3.385685 | 3.382575 | 3.388507 | -0.01035 | 0.565155 | -0.01667 | 0.492102 | 0.649638 | 0.693751 | 0.770139 | 0.799373 |
| **ko00524** | -7.60612 | -7.74924 | -7.4732 | 0.038663 | 9.888223 | 0.003404 | 0.497501 | 0.634338 | 0.674706 | 0.678119 | 0.708522 |
| **ko04075** | -6.77259 | -6.72505 | -6.81962 | -0.04614 | 4.049254 | -0.0099 | 0.494701 | 0.54015 | 0.584764 | 0.591558 | 0.626039 |
| **ko00785** | 4.492762 | 4.453934 | 4.533177 | 0.065521 | 0.862459 | 0.068322 | 0.4704 | 0.515672 | 0.568858 | 0.454309 | 0.498615 |
| **ko00460** | -8.74017 | -9.1654 | -8.31051 | 0.63062 | 5.270692 | 0.097115 | 0.442 | 0.519944 | 0.56278 | 0.149604 | 0.175656 |
| **ko00510** | 0.427958 | 0.472351 | 0.364276 | -0.07756 | 1.193953 | -0.05625 | 0.473105 | 0.506224 | 0.561012 | 0.372289 | 0.416823 |
| **ko04621** | 1.162019 | 1.177042 | 1.133444 | 0.003294 | 2.52745 | 0.00066 | 0.4987 | 0.498015 | 0.548668 | 0.857121 | 0.876057 |
| **ko00040** | 4.839578 | 4.826046 | 4.85999 | 0.028591 | 0.527681 | 0.047667 | 0.477 | 0.471412 | 0.522958 | 0.515961 | 0.554917 |
| **ko01056** | -8.48814 | -8.58169 | -8.39272 | 0.195595 | 4.258974 | 0.039381 | 0.479 | 0.462431 | 0.506298 | 0.437902 | 0.472411 |
| **ko00100** | -6.91087 | -6.85299 | -7.00127 | -0.16414 | 4.297018 | -0.03362 | 0.484103 | 0.458052 | 0.501618 | 0.571952 | 0.606702 |
| **ko00020** | 4.904741 | 4.87472 | 4.950368 | 0.052018 | 0.560652 | 0.084941 | 0.461 | 0.43716 | 0.491279 | 0.268922 | 0.308727 |
| **ko00072** | 3.572733 | 3.571701 | 3.574936 | -0.00509 | 0.864874 | -0.00249 | 0.4979 | 0.434013 | 0.490981 | 0.849094 | 0.870094 |
| **ko00362** | 2.656937 | 2.618075 | 2.707502 | 0.060431 | 0.586785 | 0.091766 | 0.4582 | 0.389541 | 0.443807 | 0.227721 | 0.265373 |
| **ko00624** | -8.49525 | -8.91352 | -8.10228 | 0.487956 | 5.285679 | 0.083523 | 0.4566 | 0.38583 | 0.431439 | 0.24101 | 0.271257 |
| **ko03450** | -2.2465 | -2.2976 | -2.15257 | -0.03793 | 2.387242 | -0.01099 | 0.4934 | 0.374836 | 0.427717 | 0.814202 | 0.838633 |
| **ko04141** | 0.511725 | 0.52212 | 0.492238 | -0.03921 | 0.845055 | -0.04413 | 0.479504 | 0.333872 | 0.389504 | 0.579142 | 0.620152 |
| **ko00720** | 5.120011 | 5.079506 | 5.182038 | 0.065334 | 0.55199 | 0.111034 | 0.4524 | 0.321209 | 0.376593 | 0.167318 | 0.20197 |
| **ko00340** | 5.361592 | 5.336298 | 5.403162 | 0.049641 | 0.698169 | 0.065449 | 0.472 | 0.297903 | 0.354107 | 0.30268 | 0.344765 |
| **ko01053** | 0.977624 | 0.891033 | 1.079886 | 0.329466 | 2.243174 | 0.120124 | 0.435913 | 0.295186 | 0.350804 | 0.077485 | 0.104995 |
| **ko04970** | -8.66229 | -8.96385 | -8.35208 | 0.603042 | 4.480757 | 0.113566 | 0.441512 | 0.260164 | 0.298323 | 0.22057 | 0.248612 |
| **ko00600** | 3.814909 | 3.936444 | 3.679518 | -0.23705 | 8.207785 | -0.03309 | 0.418 | 0.239318 | 0.292827 | 0.015176 | 0.02673 |
| **ko01040** | 3.800647 | 3.689797 | 3.920771 | 0.202404 | 0.580755 | 0.321929 | 0.3566 | 0.238842 | 0.28809 | 3.05E-05 | 0.000257 |
| **ko05130** | -8.05093 | -8.25958 | -7.8502 | 0.403844 | 5.865878 | 0.063098 | 0.463907 | 0.246238 | 0.286662 | 0.345905 | 0.378435 |
| **ko04113** | -7.4861 | -7.22736 | -7.89175 | -0.46812 | 4.177155 | -0.09093 | 0.4528 | 0.245179 | 0.283775 | 0.236291 | 0.266113 |
| **ko00906** | -3.54538 | -3.85349 | -3.23862 | 0.46977 | 2.17868 | 0.181195 | 0.410118 | 0.220233 | 0.267782 | 0.014011 | 0.024157 |
| **ko00472** | 1.975915 | 2.056682 | 1.893946 | -0.11318 | 1.682207 | -0.05261 | 0.4722 | 0.196798 | 0.249995 | 0.366105 | 0.411512 |
| **ko02030** | 5.216805 | 5.169224 | 5.287232 | 0.096725 | 0.90339 | 0.100056 | 0.4546 | 0.19885 | 0.249939 | 0.108197 | 0.139108 |
| **ko02040** | 4.393896 | 4.317824 | 4.502976 | 0.174563 | 1.241693 | 0.129154 | 0.4384 | 0.192539 | 0.244135 | 0.069568 | 0.095679 |
| **ko00052** | 5.458717 | 5.428782 | 5.499631 | 0.056653 | 0.555507 | 0.092082 | 0.4564 | 0.193581 | 0.242461 | 0.167835 | 0.202719 |
| **ko00601** | -7.20416 | -7.0492 | -7.39976 | -0.47871 | 4.208969 | -0.10507 | 0.44791 | 0.194421 | 0.235115 | 0.223771 | 0.255434 |
| **ko00965** | -8.70929 | -9.0806 | -8.32206 | 0.666658 | 4.607646 | 0.129525 | 0.432713 | 0.196215 | 0.232402 | 0.154646 | 0.178593 |
| **ko00520** | 5.323985 | 5.28483 | 5.371611 | 0.069583 | 0.524108 | 0.124718 | 0.4408 | 0.174226 | 0.221276 | 0.122944 | 0.154003 |
| **ko00621** | 2.521238 | 2.515536 | 2.538255 | -0.0498 | 10.45406 | -0.00476 | 0.490902 | 0.147942 | 0.193387 | 0.798714 | 0.8248 |
| **ko05111** | 2.471827 | 2.414114 | 2.561046 | 0.110356 | 1.091561 | 0.021549 | 0.445911 | 0.138764 | 0.185236 | 0.126904 | 0.159001 |
| **ko00121** | 5.248338 | 5.303361 | 5.171896 | -0.14898 | 1.048392 | -0.1305 | 0.4386 | 0.136197 | 0.184384 | 0.113846 | 0.144712 |
| **ko04146** | 2.77008 | 2.728867 | 2.83035 | 0.090564 | 0.572134 | 0.146582 | 0.4336 | 0.13896 | 0.183426 | 0.064498 | 0.088394 |
| **ko00195** | -8.77875 | -9.31021 | -8.23017 | 0.972772 | 5.333529 | 0.145651 | 0.411318 | 0.145805 | 0.178816 | 0.056746 | 0.071464 |
| **ko00909** | -7.91194 | -7.60592 | -8.37967 | -0.56675 | 3.820893 | -0.12735 | 0.432114 | 0.14661 | 0.177965 | 0.138646 | 0.16056 |
| **ko03020** | 5.159494 | 5.122212 | 5.216044 | 0.09944 | 0.782101 | 0.115411 | 0.44791 | 0.12885 | 0.174548 | 0.143276 | 0.176849 |
| **ko00120** | 2.08198 | 2.135596 | 2.007274 | -0.13744 | 1.049349 | -0.12306 | 0.442511 | 0.122178 | 0.168739 | 0.104511 | 0.134335 |
| **ko00250** | 5.87548 | 5.830633 | 5.93187 | 0.086853 | 0.585744 | 0.131304 | 0.4412 | 0.123642 | 0.167741 | 0.098246 | 0.127387 |
| **ko00670** | 5.840496 | 5.788977 | 5.906331 | 0.08425 | 0.604614 | 0.128795 | 0.440312 | 0.119459 | 0.163145 | 0.090979 | 0.119349 |
| **ko00900** | 5.408729 | 5.347958 | 5.482654 | 0.082971 | 0.628999 | 0.123865 | 0.4452 | 0.113755 | 0.157432 | 0.080311 | 0.107756 |
| **ko00740** | 4.861415 | 4.810514 | 4.953354 | 0.101092 | 0.598048 | 0.15341 | 0.4344 | 0.113473 | 0.156655 | 0.040022 | 0.059841 |
| **ko04112** | 5.63172 | 5.574705 | 5.703181 | 0.075852 | 0.634454 | 0.113744 | 0.4504 | 0.110711 | 0.154648 | 0.091739 | 0.120627 |
| **ko00514** | -8.35678 | -8.89033 | -7.88156 | 0.884921 | 4.724699 | 0.166049 | 0.418716 | 0.112687 | 0.142144 | 0.075093 | 0.094461 |
| **ko00190** | 3.956109 | 3.911417 | 4.016789 | 0.079303 | 0.528695 | 0.141472 | 0.4364 | 0.099938 | 0.141359 | 0.076622 | 0.102757 |
| **ko00130** | 3.337004 | 3.321394 | 3.354597 | 0.118326 | 1.059478 | 0.103197 | 0.45031 | 0.09722 | 0.140565 | 0.162281 | 0.197797 |
| **ko00860** | 4.639404 | 4.595213 | 4.705791 | 0.110254 | 0.663674 | 0.152834 | 0.4276 | 0.095378 | 0.138331 | 0.054228 | 0.077013 |
| **ko03015** | -8.57591 | -8.97778 | -8.10818 | 0.770402 | 4.465217 | 0.151162 | 0.4256 | 0.109729 | 0.138143 | 0.072768 | 0.089738 |
| **ko05322** | -8.83759 | -9.2948 | -8.34787 | 0.812341 | 4.463093 | 0.158888 | 0.417317 | 0.110111 | 0.137595 | 0.069736 | 0.085433 |
| **ko05110** | -8.67508 | -9.15602 | -8.18636 | 0.887818 | 4.63624 | 0.172645 | 0.4114 | 0.111208 | 0.136658 | 0.069804 | 0.085438 |
| **ko05145** | -8.83013 | -9.32474 | -8.32304 | 0.838124 | 4.714454 | 0.155585 | 0.422316 | 0.10575 | 0.13531 | 0.07719 | 0.095273 |
| **ko00440** | 2.590095 | 2.534543 | 2.646912 | 0.100002 | 0.558433 | 0.168962 | 0.4202 | 0.091297 | 0.130998 | 0.027165 | 0.042789 |
| **ko05012** | -6.2175 | -5.93373 | -6.69514 | -0.5969 | 4.08209 | -0.12236 | 0.4276 | 0.097862 | 0.129357 | 0.074521 | 0.096267 |
| **ko00750** | 5.222671 | 5.164274 | 5.306224 | 0.119831 | 0.553867 | 0.187761 | 0.412717 | 0.087514 | 0.128474 | 0.022908 | 0.038016 |
| **ko00630** | 4.681004 | 4.633846 | 4.747154 | 0.093005 | 0.55315 | 0.150142 | 0.4306 | 0.074732 | 0.112451 | 0.039172 | 0.058415 |
| **ko00522** | -8.96223 | -9.47368 | -8.41756 | 0.89849 | 4.572382 | 0.171638 | 0.4114 | 0.086032 | 0.106578 | 0.06229 | 0.075281 |
| **ko00903** | -8.29337 | -8.29317 | -8.29343 | -0.2898 | 6.699887 | -0.04136 | 0.474 | 0.077969 | 0.105126 | 0.43242 | 0.469323 |
| **ko03010** | 5.661364 | 5.585761 | 5.754418 | 0.112958 | 0.663373 | 0.157117 | 0.432314 | 0.065328 | 0.102253 | 0.041361 | 0.061499 |
| **ko00051** | 5.338408 | 5.296365 | 5.39851 | 0.097206 | 0.536117 | 0.168498 | 0.4244 | 0.065403 | 0.101627 | 0.042736 | 0.062708 |
| **ko00790** | 5.20381 | 5.150545 | 5.281307 | 0.102074 | 0.576223 | 0.162693 | 0.427115 | 0.062462 | 0.098198 | 0.033657 | 0.051887 |
| **ko00471** | 6.119222 | 6.043864 | 6.206347 | 0.111595 | 0.644031 | 0.166813 | 0.425515 | 0.056567 | 0.091036 | 0.035449 | 0.054318 |
| **ko03030** | 5.326209 | 5.258414 | 5.410574 | 0.097313 | 0.589206 | 0.146587 | 0.432314 | 0.055832 | 0.090148 | 0.037876 | 0.057237 |
| **ko00401** | -8.94107 | -9.52227 | -8.36261 | 1.038885 | 4.571553 | 0.200582 | 0.4018 | 0.067908 | 0.089048 | 0.034109 | 0.045124 |
| **ko00590** | -8.92913 | -9.502 | -8.35546 | 0.937786 | 4.484356 | 0.183628 | 0.4068 | 0.066801 | 0.087834 | 0.033198 | 0.044424 |
| **ko00622** | -7.76179 | -7.6381 | -7.86005 | -0.4882 | 8.832666 | -0.0588 | 0.4616 | 0.058771 | 0.086785 | 0.295633 | 0.331207 |
| **ko00310** | 2.612272 | 2.540245 | 2.714266 | 0.142248 | 0.55909 | 0.222259 | 0.3996 | 0.053638 | 0.085902 | 0.004069 | 0.009848 |
| **ko00980** | -8.90348 | -9.45833 | -8.34207 | 1.024518 | 4.708681 | 0.188494 | 0.405119 | 0.060909 | 0.082683 | 0.028279 | 0.03883 |
| **ko00943** | -8.90184 | -9.44037 | -8.33715 | 1.021195 | 4.555637 | 0.203068 | 0.4046 | 0.060957 | 0.082511 | 0.029581 | 0.039775 |
| **ko00523** | -8.3598 | -8.46276 | -8.26292 | -0.22698 | 7.089811 | -0.02952 | 0.4828 | 0.053603 | 0.079245 | 0.521743 | 0.557563 |
| **ko03440** | 5.648789 | 5.578703 | 5.734807 | 0.11006 | 0.590294 | 0.175975 | 0.419916 | 0.04613 | 0.077596 | 0.028086 | 0.045156 |
| **ko05010** | -8.94696 | -9.49331 | -8.37793 | 1.054267 | 4.560086 | 0.199238 | 0.3998 | 0.056712 | 0.074779 | 0.03388 | 0.044279 |
| **ko03060** | 5.581375 | 5.515282 | 5.662 | 0.11368 | 0.589464 | 0.174171 | 0.418116 | 0.043575 | 0.074039 | 0.029396 | 0.046693 |
| **ko04962** | -8.9877 | -9.57145 | -8.38306 | 1.035419 | 4.542038 | 0.19936 | 0.39952 | 0.054798 | 0.073955 | 0.029698 | 0.039646 |
| **ko00400** | 5.378842 | 5.318382 | 5.449944 | 0.110676 | 0.635157 | 0.158472 | 0.427914 | 0.042521 | 0.072991 | 0.031084 | 0.048967 |
| **ko00300** | 5.814592 | 5.762967 | 5.889985 | 0.112955 | 0.62724 | 0.165498 | 0.425 | 0.041453 | 0.071586 | 0.042563 | 0.06276 |
| **ko00196** | -8.81444 | -9.35596 | -8.20915 | 0.993976 | 4.750099 | 0.183606 | 0.4012 | 0.052233 | 0.071329 | 0.030749 | 0.041554 |
| **ko04512** | -8.97857 | -9.50772 | -8.40864 | 0.995368 | 4.564021 | 0.190611 | 0.406719 | 0.050545 | 0.06997 | 0.03174 | 0.042634 |
| **ko04614** | -8.92189 | -9.48633 | -8.32973 | 1.010117 | 4.59499 | 0.192441 | 0.404519 | 0.050469 | 0.068483 | 0.030036 | 0.040096 |
| **ko03040** | -8.87004 | -9.45713 | -8.26438 | 1.013878 | 4.596097 | 0.195733 | 0.405919 | 0.041138 | 0.059254 | 0.020132 | 0.029785 |
| **ko00363** | -8.75528 | -9.33207 | -8.1496 | 1.143356 | 5.311918 | 0.176198 | 0.393121 | 0.040313 | 0.058579 | 0.030154 | 0.039649 |
| **ko00380** | 2.554326 | 2.511157 | 2.616165 | 0.07985 | 0.859769 | 0.014892 | 0.454109 | 0.032955 | 0.057428 | 0.264033 | 0.304376 |
| **ko03050** | -5.04671 | -5.49916 | -4.37643 | 0.888503 | 3.283759 | 0.228329 | 0.3836 | 0.034866 | 0.056211 | 0.001165 | 0.003412 |
| **ko04020** | -8.58832 | -9.2678 | -7.92747 | 1.181944 | 4.552528 | 0.224008 | 0.389122 | 0.040896 | 0.053925 | 0.017369 | 0.023852 |
| **ko00642** | -8.97127 | -9.53326 | -8.35122 | 1.052284 | 4.62741 | 0.195705 | 0.3994 | 0.037058 | 0.052662 | 0.023631 | 0.03266 |
| **ko03430** | 5.812583 | 5.742743 | 5.898363 | 0.120367 | 0.591131 | 0.195254 | 0.417 | 0.027716 | 0.050875 | 0.020595 | 0.0351 |
| **ko03013** | 1.047131 | 0.974097 | 1.130547 | 0.124341 | 0.645879 | 0.18221 | 0.421916 | 0.027465 | 0.048433 | 0.020308 | 0.033317 |
| **ko00071** | 3.665437 | 3.591691 | 3.758746 | 0.14681 | 0.501219 | 0.246492 | 0.3806 | 0.025858 | 0.047886 | 0.000646 | 0.002535 |
| **ko04910** | 1.76653 | 1.694903 | 1.860348 | 0.147475 | 0.624903 | 0.211008 | 0.407119 | 0.020068 | 0.039003 | 0.010194 | 0.019896 |
| **ko04142** | -8.87037 | -9.46609 | -8.21679 | 1.1478 | 4.873841 | 0.209113 | 0.393121 | 0.024944 | 0.038648 | 0.015712 | 0.023238 |
| **ko00970** | 5.760277 | 5.684799 | 5.851332 | 0.125267 | 0.632597 | 0.182732 | 0.419716 | 0.01862 | 0.036546 | 0.014798 | 0.026866 |
| **ko00240** | 5.271119 | 5.192921 | 5.366011 | 0.119368 | 0.587196 | 0.192052 | 0.416117 | 0.018342 | 0.036049 | 0.010625 | 0.02086 |
| **ko05120** | 2.429552 | 2.350093 | 2.518627 | 0.132891 | 0.653494 | 0.186586 | 0.415717 | 0.017895 | 0.035537 | 0.015068 | 0.027053 |
| **ko00330** | 4.715401 | 4.651993 | 4.802926 | 0.113835 | 0.548604 | 0.18689 | 0.4126 | 0.016171 | 0.03274 | 0.010209 | 0.020093 |
| **ko00730** | 5.816964 | 5.748475 | 5.896635 | 0.137811 | 0.656387 | 0.199888 | 0.4154 | 0.014791 | 0.031056 | 0.013252 | 0.024759 |
| **ko00770** | 5.932224 | 5.863143 | 6.009049 | 0.127444 | 0.592132 | 0.200571 | 0.408718 | 0.014547 | 0.030473 | 0.011431 | 0.022056 |
| **ko00710** | 5.778594 | 5.716696 | 5.854861 | 0.117544 | 0.561313 | 0.186711 | 0.4154 | 0.014471 | 0.030374 | 0.012616 | 0.023739 |
| **ko00521** | 5.824429 | 5.767998 | 5.888657 | 0.118917 | 0.619079 | 0.181458 | 0.4162 | 0.013811 | 0.029515 | 0.025194 | 0.041216 |
| **ko00260** | 5.22973 | 5.153172 | 5.328569 | 0.122168 | 0.533535 | 0.212097 | 0.4076 | 0.013813 | 0.029296 | 0.005141 | 0.011985 |
| **ko03420** | 4.689459 | 4.61405 | 4.786134 | 0.138047 | 0.64133 | 0.193538 | 0.4118 | 0.013706 | 0.029262 | 0.012522 | 0.023662 |
| **ko00253** | -8.90083 | -9.4924 | -8.24629 | 1.194284 | 4.844165 | 0.205517 | 0.391722 | 0.018112 | 0.028726 | 0.019063 | 0.026256 |
| **ko00061** | 5.80596 | 5.73586 | 5.894248 | 0.127292 | 0.605462 | 0.197101 | 0.411918 | 0.01148 | 0.025814 | 0.009573 | 0.019329 |
| **ko04626** | 2.812593 | 2.732761 | 2.909241 | 0.140398 | 0.605125 | 0.213263 | 0.403719 | 0.01141 | 0.025311 | 0.005234 | 0.012063 |
| **ko00500** | 5.374759 | 5.314915 | 5.443593 | 0.117733 | 0.557225 | 0.192909 | 0.412 | 0.008956 | 0.021376 | 0.009767 | 0.019432 |
| **ko00920** | 4.836697 | 4.778698 | 4.912234 | 0.13114 | 0.525955 | 0.23028 | 0.3956 | 0.008842 | 0.020953 | 0.004345 | 0.010524 |
| **ko00830** | -6.49396 | -4.95398 | -7.2136 | -1.12617 | 8.43905 | -0.15127 | 0.411718 | 0.00947 | 0.019947 | 0.021146 | 0.033123 |
| **ko03022** | -8.79895 | -9.43873 | -8.11717 | 1.238082 | 4.904617 | 0.218817 | 0.3846 | 0.010564 | 0.01919 | 0.006851 | 0.011748 |
| **ko00511** | 5.384988 | 5.504214 | 5.287903 | -0.2418 | 1.032904 | -0.22072 | 0.4032 | 0.007932 | 0.018649 | 0.005123 | 0.011529 |
| **ko05410** | -6.80864 | -6.32681 | -7.41855 | -0.99332 | 3.737927 | -0.2274 | 0.3776 | 0.011353 | 0.018435 | 0.002972 | 0.005464 |
| **ko00290** | 6.270033 | 6.200577 | 6.357754 | 0.143482 | 0.608372 | 0.22427 | 0.407 | 0.006931 | 0.017654 | 0.006788 | 0.014846 |
| **ko03018** | 4.335187 | 4.246052 | 4.438125 | 0.13991 | 0.569561 | 0.235066 | 0.396 | 0.006307 | 0.016382 | 0.003339 | 0.008728 |
| **ko00281** | 1.684414 | 1.860333 | 1.415829 | -0.43941 | 6.725107 | -0.06737 | 0.4184 | 0.00653 | 0.01609 | 0.011459 | 0.021912 |
| **ko00550** | 5.975606 | 5.896184 | 6.068391 | 0.145236 | 0.61382 | 0.223639 | 0.4052 | 0.005942 | 0.015705 | 0.005081 | 0.011933 |
| **ko00591** | 1.735967 | 2.59283 | -5.79216 | -0.82182 | 10.63372 | -0.08784 | 0.387 | 0.006389 | 0.015353 | 0.001052 | 0.003289 |
| **ko00311** | 0.119541 | -0.09319 | 0.37239 | 0.423814 | 1.558944 | 0.244518 | 0.384 | 0.005845 | 0.014283 | 0.00048 | 0.002058 |
| **ko00620** | 5.37312 | 5.301301 | 5.462545 | 0.131161 | 0.541712 | 0.230703 | 0.396321 | 0.00508 | 0.01388 | 0.003655 | 0.009338 |
| **ko05142** | -8.66908 | -9.35529 | -7.93409 | 1.423889 | 5.322872 | 0.243919 | 0.380724 | 0.007616 | 0.01364 | 0.005009 | 0.008819 |
| **ko00312** | 3.528922 | 3.421227 | 3.650431 | 0.23846 | 0.991148 | 0.213384 | 0.3942 | 0.004824 | 0.013611 | 0.002282 | 0.006685 |
| **ko00540** | 4.210823 | 4.277062 | 4.107037 | -0.1993 | 1.268641 | -0.14679 | 0.4296 | 0.004816 | 0.013171 | 0.021655 | 0.035769 |
| **ko00270** | 5.509998 | 5.433804 | 5.603052 | 0.141282 | 0.559228 | 0.232579 | 0.396321 | 0.004573 | 0.012927 | 0.003157 | 0.008443 |
| **ko00562** | 2.819773 | 2.750861 | 2.888675 | 0.142542 | 0.560947 | 0.221652 | 0.398 | 0.004079 | 0.011833 | 0.005133 | 0.011771 |
| **ko00030** | 5.96246 | 5.893589 | 6.046135 | 0.143314 | 0.565383 | 0.234465 | 0.396521 | 0.003972 | 0.011693 | 0.00435 | 0.010528 |
| **ko05150** | -1.30005 | -1.5608 | -0.97855 | 0.594809 | 2.238995 | 0.237792 | 0.3804 | 0.003607 | 0.009938 | 0.000291 | 0.001411 |
| **ko02060** | 4.326128 | 4.182145 | 4.46999 | 0.250435 | 0.944527 | 0.246401 | 0.390122 | 0.002879 | 0.009564 | 0.000742 | 0.002871 |
| **ko00230** | 4.924373 | 4.839204 | 5.024123 | 0.137622 | 0.539765 | 0.235141 | 0.3948 | 0.002811 | 0.00918 | 0.001488 | 0.004824 |
| **ko00450** | 5.185641 | 5.112213 | 5.273202 | 0.136453 | 0.499036 | 0.252462 | 0.384323 | 0.002816 | 0.009104 | 0.001232 | 0.004151 |
| **ko00650** | 4.633036 | 4.559209 | 4.725174 | 0.157115 | 0.511881 | 0.28078 | 0.378524 | 0.002841 | 0.009088 | 0.000992 | 0.003469 |
| **ko01055** | 6.206786 | 6.116961 | 6.30618 | 0.146516 | 0.623249 | 0.218915 | 0.39932 | 0.002706 | 0.008995 | 0.00352 | 0.009168 |
| **ko00760** | 5.227634 | 5.136349 | 5.345894 | 0.151535 | 0.568668 | 0.244834 | 0.387722 | 0.002412 | 0.008344 | 0.000785 | 0.00301 |
| **ko00680** | 4.223548 | 4.156055 | 4.302962 | 0.137797 | 0.551379 | 0.233386 | 0.39792 | 0.002311 | 0.008043 | 0.002974 | 0.008008 |
| **ko00140** | 0.285013 | 0.404466 | 0.141198 | -0.33951 | 1.669452 | -0.15607 | 0.4038 | 0.002294 | 0.007843 | 0.009901 | 0.019332 |
| **ko00623** | -7.46137 | -6.97871 | -7.82474 | -1.07774 | 9.570074 | -0.1194 | 0.424715 | 0.002505 | 0.007108 | 0.040828 | 0.055458 |
| **ko03410** | 4.917707 | 4.821891 | 5.0216 | 0.156518 | 0.562666 | 0.265836 | 0.3898 | 0.001746 | 0.006593 | 0.001136 | 0.003905 |
| **ko00561** | 4.285039 | 4.217409 | 4.36891 | 0.134093 | 0.55044 | 0.22373 | 0.3982 | 0.001546 | 0.006026 | 0.002167 | 0.006264 |
| **ko00473** | 5.790448 | 5.688365 | 5.913215 | 0.180041 | 0.54346 | 0.302019 | 0.369926 | 0.001101 | 0.004784 | 0.000346 | 0.001645 |
| **ko04122** | 5.280608 | 5.203492 | 5.385915 | 0.156502 | 0.567585 | 0.251349 | 0.387 | 0.000921 | 0.004228 | 0.000737 | 0.002869 |
| **ko00910** | 4.518082 | 4.443515 | 4.603719 | 0.153958 | 0.497209 | 0.286678 | 0.3736 | 0.000918 | 0.004213 | 0.000383 | 0.001755 |
| **ko00053** | 3.366193 | 3.272854 | 3.4679 | 0.235621 | 0.797384 | 0.25919 | 0.3774 | 0.000736 | 0.003694 | 0.000453 | 0.001971 |
| **ko03070** | 4.754978 | 4.657719 | 4.864138 | 0.172562 | 0.531972 | 0.297659 | 0.3712 | 0.00074 | 0.003624 | 0.000136 | 0.000817 |
| **ko00010** | 5.339636 | 5.24932 | 5.437925 | 0.167122 | 0.52732 | 0.290553 | 0.371926 | 0.000735 | 0.003623 | 0.000401 | 0.00181 |
| **ko03008** | 0.922044 | 0.805893 | 1.05197 | 0.205077 | 0.604541 | 0.312886 | 0.367327 | 0.000739 | 0.003485 | 0.000196 | 0.001021 |
| **ko04974** | -0.29361 | -0.07147 | -0.61749 | -0.62498 | 1.976775 | -0.26084 | 0.3608 | 0.000914 | 0.003324 | 0.000102 | 0.000624 |
| **ko04144** | -7.58482 | -8.52702 | -6.88062 | 1.369448 | 4.461237 | 0.27588 | 0.368126 | 0.000778 | 0.002555 | 0.00057 | 0.001522 |
| **ko00480** | 4.007089 | 3.915084 | 4.122436 | 0.213756 | 0.668757 | 0.291696 | 0.3602 | 0.000388 | 0.002383 | 5.64E-05 | 0.000413 |
| **ko01051** | 7.460482 | 7.38874 | 7.554675 | 0.190418 | 0.663389 | 0.258554 | 0.3864 | 0.000337 | 0.002163 | 0.001402 | 0.004637 |
| **ko05100** | -1.42632 | -2.04892 | -0.98725 | 1.261438 | 3.798136 | 0.298433 | 0.363727 | 0.000436 | 0.002095 | 4.76E-05 | 0.000374 |
| **ko00660** | 6.00168 | 5.899244 | 6.111954 | 0.174937 | 0.601732 | 0.273635 | 0.3808 | 0.000275 | 0.001856 | 0.000317 | 0.001553 |
| **ko00626** | -8.28333 | -9.20154 | -7.27484 | 1.979128 | 9.285187 | 0.232878 | 0.3546 | 0.000256 | 0.00127 | 0.000179 | 0.000712 |
| **ko00364** | -6.44608 | -3.21575 | -7.41933 | -2.601 | 7.810096 | -0.32642 | 0.370726 | 0.000191 | 0.001162 | 0.000418 | 0.001615 |
| **ko01057** | -7.52219 | -6.33139 | -8.35832 | -2.0067 | 4.485739 | -0.39969 | 0.3104 | 0.000314 | 0.001117 | 2.82E-06 | 2.82E-05 |
| **ko00410** | -4.39881 | 3.644253 | -6.36328 | -0.91993 | 11.41531 | -0.11874 | 0.3714 | 0.000163 | 0.00108 | 0.000429 | 0.001577 |
| **ko04210** | -1.07256 | -0.83526 | -1.41016 | -0.66397 | 2.146582 | -0.26598 | 0.3712 | 9.59E-05 | 0.000727 | 0.000112 | 0.00067 |
| **ko02020** | 3.859482 | 3.744238 | 4.000122 | 0.219065 | 0.602447 | 0.325177 | 0.346131 | 5.34E-05 | 0.000497 | 7.04E-06 | 8.84E-05 |
| **ko00531** | 3.580197 | 3.877967 | 3.336742 | -0.56677 | 1.575624 | -0.3148 | 0.353729 | 4.23E-05 | 0.000363 | 1.20E-05 | 0.000133 |
| **ko00564** | 4.415319 | 4.319492 | 4.519968 | 0.194109 | 0.519826 | 0.350434 | 0.343 | 2.10E-05 | 0.000229 | 1.54E-05 | 0.000153 |
| **ko00361** | 1.307904 | 1.14768 | 1.498803 | 0.370549 | 1.051169 | 0.329323 | 0.35073 | 9.21E-06 | 0.000128 | 7.59E-06 | 9.39E-05 |
| **ko00633** | 3.712952 | 3.510708 | 3.877707 | 0.336618 | 0.86326 | 0.354694 | 0.340132 | 4.93E-06 | 7.89E-05 | 2.43E-06 | 4.09E-05 |
| **ko00960** | 2.838557 | 3.507089 | -6.57443 | -1.6252 | 11.14175 | -0.2427 | 0.357129 | 2.21E-06 | 3.79E-05 | 4.11E-05 | 0.000272 |
| **ko02010** | 4.84507 | 4.730388 | 4.964458 | 0.231158 | 0.559444 | 0.391283 | 0.328 | 1.75E-06 | 3.13E-05 | 1.06E-06 | 2.10E-05 |
| **ko00791** | -6.1264 | -0.69427 | -7.39188 | -3.63697 | 8.102765 | -0.45637 | 0.333 | 1.10E-06 | 1.96E-05 | 5.71E-06 | 6.50E-05 |
| **ko00643** | -5.6182 | 0.464836 | -7.13903 | -3.60596 | 8.415247 | -0.46302 | 0.3146 | 6.01E-08 | 1.65E-06 | 1.27E-07 | 3.46E-06 |
| **ko00983** | -5.9725 | 4.987044 | -7.24819 | -3.88607 | 12.43949 | -0.4361 | 0.3138 | 2.97E-08 | 1.04E-06 | 4.49E-07 | 8.18E-06 |
| **ko00625** | -6.62262 | 3.028075 | -7.7005 | -4.22582 | 10.47242 | -0.51914 | 0.302539 | 1.27E-08 | 3.56E-07 | 1.99E-07 | 3.68E-06 |
| **ko05131** | -3.51454 | -7.97347 | -1.48655 | 5.367682 | 7.455381 | 0.676474 | 0.2346 | 1.58E-14 | 2.75E-12 | 9.53E-15 | 1.68E-12 |
